# Graph neural networks for single-cell omics data: a review of approaches and applications

**DOI:** 10.1093/bib/bbaf109

**Published:** 2025-03-17

**Authors:** Sijie Li, Heyang Hua, Shengquan Chen

**Affiliations:** School of Mathematical Sciences and The Key Laboratory of Pure Mathematics and Combinatorics, Ministry of Education (LPMC), Nankai University, No. 94 Weijin Road, Nankai District, Tianjin 300071, China; School of Mathematical Sciences and The Key Laboratory of Pure Mathematics and Combinatorics, Ministry of Education (LPMC), Nankai University, No. 94 Weijin Road, Nankai District, Tianjin 300071, China; School of Mathematical Sciences and The Key Laboratory of Pure Mathematics and Combinatorics, Ministry of Education (LPMC), Nankai University, No. 94 Weijin Road, Nankai District, Tianjin 300071, China

**Keywords:** graph neutral networks, single-cell, epigenomics, transcriptomics, proteomics

## Abstract

Rapid advancement of sequencing technologies now allows for the utilization of precise signals at single-cell resolution in various omics studies. However, the massive volume, ultra-high dimensionality, and high sparsity nature of single-cell data have introduced substantial difficulties to traditional computational methods. The intricate non-Euclidean networks of intracellular and intercellular signaling molecules within single-cell datasets, coupled with the complex, multimodal structures arising from multi-omics joint analysis, pose significant challenges to conventional deep learning operations reliant on Euclidean geometries. Graph neural networks (GNNs) have extended deep learning to non-Euclidean data, allowing cells and their features in single-cell datasets to be modeled as nodes within a graph structure. GNNs have been successfully applied across a broad range of tasks in single-cell data analysis. In this survey, we systematically review 107 successful applications of GNNs and their six variants in various single-cell omics tasks. We begin by outlining the fundamental principles of GNNs and their six variants, followed by a systematic review of GNN-based models applied in single-cell epigenomics, transcriptomics, spatial transcriptomics, proteomics, and multi-omics. In each section dedicated to a specific omics type, we have summarized the publicly available single-cell datasets commonly utilized in the articles reviewed in that section, totaling 77 datasets. Finally, we summarize the potential shortcomings of current research and explore directions for future studies. We anticipate that this review will serve as a guiding resource for researchers to deepen the application of GNNs in single-cell omics.

## Introduction

The advent of bulk sequencing technologies such as ribonucleic acid (RNA) sequencing (RNA-seq) [1, 2], assay of transposase accessible chromatin sequencing (ATAC-seq) [3, 4], and bisulfite sequencing (BS-seq) [5, 6] has significantly advanced the investigation of differential gene expression [7], detection of chromatin accessibility changes between different cell states [8], and interpretation of biological processes such as cellular development and aging through deoxyribonucleic acid (DNA) methylation, providing insights into the mechanisms and etiology of diseases like cancer and leukemia [8–10]. However, bulk sequencing technologies measure the average signals of cells in the whole tissue, thus failing to capture the heterogeneity among individual cells [11]. Rapid revolution in sequencing technology has facilitated the emergence of sequencing technologies at single-cell resolution, including single-cell RNA sequencing (scRNA-seq) [12], spatial resolved transcriptomics profiling at single-cell resolution [13–15], single-cell chromatin accessibility sequencing (scCAS) [16–20], single-cell DNA methylation (scDNAm) [21], single-cell Hi-C [22], single-cell proteomics sequencing [23, 24], and single-cell multi-omics sequencing [25–32].

Single-cell data provides a revolutionary scale and high resolution that bulk data lacked in the past, allowing for the characterization of cell populations at an unprecedented level, especially the heterogeneity of cell subtypes and rare cell types, as well as various cell states [33, 34]. It has been widely utilized for clustering and classification to identify cell populations and reported to shed light on cell type heterogeneity through various downstream analyses [35–39], facilitating exploration of complex phenotypes and potential gene regulatory mechanisms. Single-cell omics data also enables the decipherment of differentiation trajectories [40], inference of cell–cell communications [41] and Granger causal relationship [42], elucidation of the diversity of cell phenotypes within the tumor microenvironment and inference of the tumor lineages [43, 44], play a crucial role in uncovering cancer biology.

**Figure 1:**
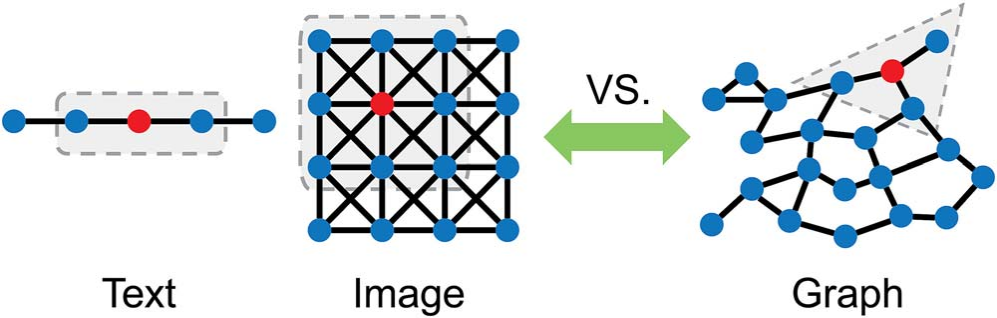
1D and 2D data versus graph data. 1D data (e.g. text) is Euclidean, with a fixed sequence that allows for linear processing, such as sequential convolutions over the data. 2D data (e.g. images) is also Euclidean, with a grid-like structure that allows for spatial convolution operations to capture local features like edges and textures. Unlike one- and 2D data, graph data is non-Euclidean. It does not have a fixed structure or linear order, and its relationships are defined by the graph’s topology.

Although single-cell data provides a high-resolution perspective for researching cell heterogeneity, modeling single-cell data presents significant challenges due to the unique characteristics of these omics datasets. Single-cell data is high-dimensional, sparse, and often noisy, making it difficult to directly apply traditional statistical and machine learning methods. Furthermore, the complex biological relationships between different types of omics data (e.g. gene expression, chromatin accessibility, DNA methylation) are difficult to model using traditional machine learning approaches. These challenges necessitate the development of novel computational approaches, one feasible method being to model single-cell data as graphs. Actually, both cells and features in single-cell data can be regarded as nodes, potentially connected by various types of links, such as cell–cell communications [45–47] and gene–gene regulatory interactions [48]. Such graph structure alters the fundamental assumption of traditional machine learning algorithms that the random variables are independent [49]. However, the non-Euclidean structure of single-cell data, unlike the grid-like structure of 1D and 2D data (Fig. 1), poses challenges to deep learning operations such as convolution, which are traditionally designed for Euclidean space [50]. Emerging graph neural networks (GNNs) have extended deep learning to non-Euclidean structure data [51] and demonstrated promising potential for the exploration of single-cell data.

GNNs [52, 53] are end-to-end deep learning neural network models that have recently made remarkable progress. Several variants of GNNs, including graph convolutional networks (GCNs) [54], GraphSAGE [55], graph attention networks (GATs) [56], graph transformer networks (GTNs) [57], graph autoencoders (GAEs) [5, 8] and variational graph autoencoders (VGAEs) [58] have been extensively applied in the field of bioinformatics [59] and proven highly effective across various applications in diverse areas, such as disease prediction [60, 61] and drug discovery [62].

For single-cell omics analysis, GNN-based models can learn low-dimensional representations integrated with topological information from various graphs such as cell–cell [63], cell-gene [64], and gene–gene [65] graphs. GNN models have exhibited remarkable capabilities in a wide range of single-cell omics analysis, including preprocessing tasks such as enhancement, imputation, and batch effect correction of the single-cell data, as well as key tasks like cell clustering and classification to identify cell types [66–70]. Besides, GNN models also contribute to gaining valuable biological insights by modeling single-cell data for downstream analyses such as gene regulatory network reconstruction and intercellular communication. Notably, GNN-based approaches for single-cell omics have demonstrated superior performance compared to traditional methods, including those specifically designed for single-cell data analysis and general traditional machine learning techniques (Supplementary Text S1). Therefore, summarizing the application of GNN models in single-cell omics is essential for advancing single-cell data analysis. Lazaros *et al.* have provided a foundational reference for the application of GNN-based models in single-cell data [71]. However, they mainly focus on applications for scRNA-seq and spatial transcriptomics (ST) data, with limited discussion on single-cell epigenomics, such as scCAS data and scDNAm data. Additionally, their work did not comprehensively consider all main GNN variants, for example, graph transformer networks are not adequately covered in their study. Furthermore, dozens of novel GNN-based models have not been included, partially due to their timeliness.

Our comprehensive survey provides a concise overview of the principles behind various variants of GNN models and thoroughly explores their notable applications in single-cell omics analyses, keeping abreast of their latest related developments. Firstly, we elaborate on the principles of GNNs and introduce six variants of GNNs that play a significant role in single-cell omics analysis, including GCNs, GraphSAGE, GATs, GTNs, GAEs, and VGAEs. Then, we investigate their key applications across multiple modalities of single-cell data, including scCAS data, scDNAm data, scHi-C data, scRNA-seq data, spatially resolved transcriptomics (SRT) data, and single-cell multi-omics data. We review all the GNN models designed in >107 publications, focusing on their applications in various tasks such as data imputation, dimensionality reduction, cell type identification, cell type deconvolution, spatial domain identification, and multi-omics integration. In each omics-specific chapter, we have also summarized the publicly available single-cell datasets commonly used in the GNN-based articles reviewed in that section, with a total of 77 commonly used datasets collected throughout the manuscript. Finally, we elucidate the inherent challenges for each single-cell modality data and discuss the pros and cons of the GNN-based single-cell modeling methodologies. We anticipate that this survey will provide valuable guidance and reference for the future applications of GNNs and the development of computational methods for single-cell data.

## Principles of graph neural networks: a brief overview

In this section, we provide a concise introduction to GNN and six commonly used GNN variants that we captured during our review process, enabling quick reference for readers. We recommend [51, 72] for further extensive review of GNNs.

A graph can be represented as $\mathcal{G}=\left(\mathcal{V},\mathcal{E}\right)$, where $\mathcal{V}$ and $\mathcal{E}$ are the set of nodes and edges in the graph, respectively. The adjacency matrix A of $\mathcal{G}$ is a ${N}_v\times{N}_v$ matrix, where ${N}_v=\left|\mathcal{V}\right|$ is the number of nodes. If there exists an edge from node $v$ to node $u$, i.e. ${e}_{vu}\in \mathcal{E}$, then $\mathbf{A}\left(v,u\right)$ represents the weight of ${e}_{vu}$; otherwise $\mathbf{A}\left(v,u\right)=0$. For undirected graphs, $\mathbf{A}\left(v,u\right)=1$ if ${e}_{vu}\in \mathcal{E}$ and A is a symmetric matrix, which does not hold necessarily for directed graphs. A graph is homogeneous if it contains only one type of nodes and one type of edges; otherwise, it is classified as a heterogeneous graph. The edge features ${\mathbf{e}}_{vu}$ represents the attribute information of edge ${e}_{vu}$. Edge features can be discrete, such as binary values indicating the existence of an edge, or continuous, representing the strength of the relationship between nodes. Additionally, they can be both unidimensional, as previously mentioned, or multidimensional, capturing the complex relationships between nodes. We have summarized the frequently used symbols in this article and their corresponding meanings in Table 1 for conciseness.

**Table 1:** Notations used in this study.

Notations	Descriptions
$\mathcal{G}$	A graph
$\mathcal{V}$	The set of nodes in $\mathcal{G}$
$\mathcal{E}$	The set of edges in $\mathcal{G}$
$v$	A node $v\in \mathcal{V}$
${e}_{vu}$	Edge ${e}_{vu}\in \mathcal{E}$ between node $v$ and node $u$
A	The adjacency matrix of $\mathcal{G}$
${\mathbf{A}}^T$	The transpose of matrix A
${N}_v$	The number of nodes in $\mathcal{G}$
${N}_e$	The number of edges in $\mathcal{G}$
$\mathcal{N}(v)$	The neighborhood of node $v$, $\mathcal{N}(v)=\left\{\ u\in \mathcal{V}|\left(v,u\right)\in \mathcal{E}\ \right\}$
${\mathbb{R}}^n$	$n$ -dimensional Euclidean space
${\boldsymbol{y}}_v$	The feature of node $v$
${\mathbf{y}}_{co\left[v\right]}$	The features of the edges of node $v$
${\mathbf{e}}_{vu}$	The features of the edge ${e}_{vu}$
${\mathbf{y}}_{\mathcal{N}(v)}$	The features of the nodes in $\mathcal{N}(v)$
${\boldsymbol{h}}_v$	The hidden state of node $v$
${\boldsymbol{h}}_v^{(t)}$	The hidden state of node $v$ at the $t$-th iteration
${\mathbf{h}}_{\mathcal{N}(v)}$	The hidden states of the nodes in $\mathcal{N}(v)$
${\mathbf{h}}_{\mathcal{N}\left(\mathcal{v}\right)}^{(t)}$	The hidden states of the nodes in $\mathcal{N}(v)$ at the *t*-th iteration
$\left|\cdotp \right|$	The number of elements in a set
$\sigma \left(\cdotp \right)$	The activation function
${\mathbf{I}}_N$	The $N\times N$ identity matrix
**D**	The degree matrix of $\mathcal{G},{\mathbf{D}}_{ii}={\sum}_j{\mathbf{A}}_{ij}$
$\parallel$	The concatenation operator

The GNN model was first introduced by Gori *et al.* [52] and Scarselli *et al.* [53]. GNN aims to extend neural network approaches to handle various types of graph inputs, such as directed and undirected graphs. It learns the representation of each node $v$, denoted as the hidden state ${\boldsymbol{h}}_v\in{\mathbb{R}}^s$, leveraging the neighborhood of $v$. GNN mainly consists of the forward and backward process. The forward process involves a parametric function ${f}_{\boldsymbol{w}}$, known as the transition function, where $\boldsymbol{w}$ represents a set of parameters. ${f}_{\boldsymbol{w}}$ aggregates information from the edges connected to a node and its neighboring nodes to generate the hidden state of the node. However, obtaining the nodes’ hidden states is not the ultimate aim. The goal of a GNN is not merely to generate the hidden states of nodes. These hidden states can be seen as feature representations of nodes, but their real significance lies in their application to practical tasks. To achieve specific tasks, such as node classification, the GNN uses a local output function ${g}_{\boldsymbol{w}}$, which is typically a learnable transformation function, to convert these hidden states into final output results, such as node labels. The states of nodes and outputs are iteratively computed as follows:


(1) \begin{equation*} {\boldsymbol{h}}_v^{\left(t+1\right)}={f}_{\boldsymbol{w}}\left({\boldsymbol{y}}_v,{\mathbf{y}}_{co\left[v\right]},{\mathbf{h}}_{\mathcal{N}(v)}^{(t)},{\mathbf{y}}_{\mathcal{N}(v)}\right) \end{equation*}

(2) \begin{equation*} {\displaystyle \begin{array}{c}{\boldsymbol{o}}_v^{(t)}={g}_{\boldsymbol{w}}\left({\boldsymbol{h}}_v^{(t)},{\boldsymbol{y}}_v\right)\end{array}} \end{equation*}

where ${\boldsymbol{o}}_v^{(t)}$ denotes the outputs of the $i$-th iteration and ${\mathbf{y}}_{co\left[v\right]}$ represents the features of the edges of node $v$. Given a threshold ${\varepsilon}_f$ and a norm $\left\Vert \cdotp \right\Vert$, the aforementioned iteration stops when $\big\Vert{\boldsymbol{h}}_v^{(t)}-{\boldsymbol{h}}_v^{\left(t-1\right)}\big\Vert <{\varepsilon}_f$. GNN then calculates the gradient in the backward process to update the parameter set $\boldsymbol{w}$. GNN iteratively performs the forward and backward processes until a specified stopping criterion is reached.

**Figure 2:**
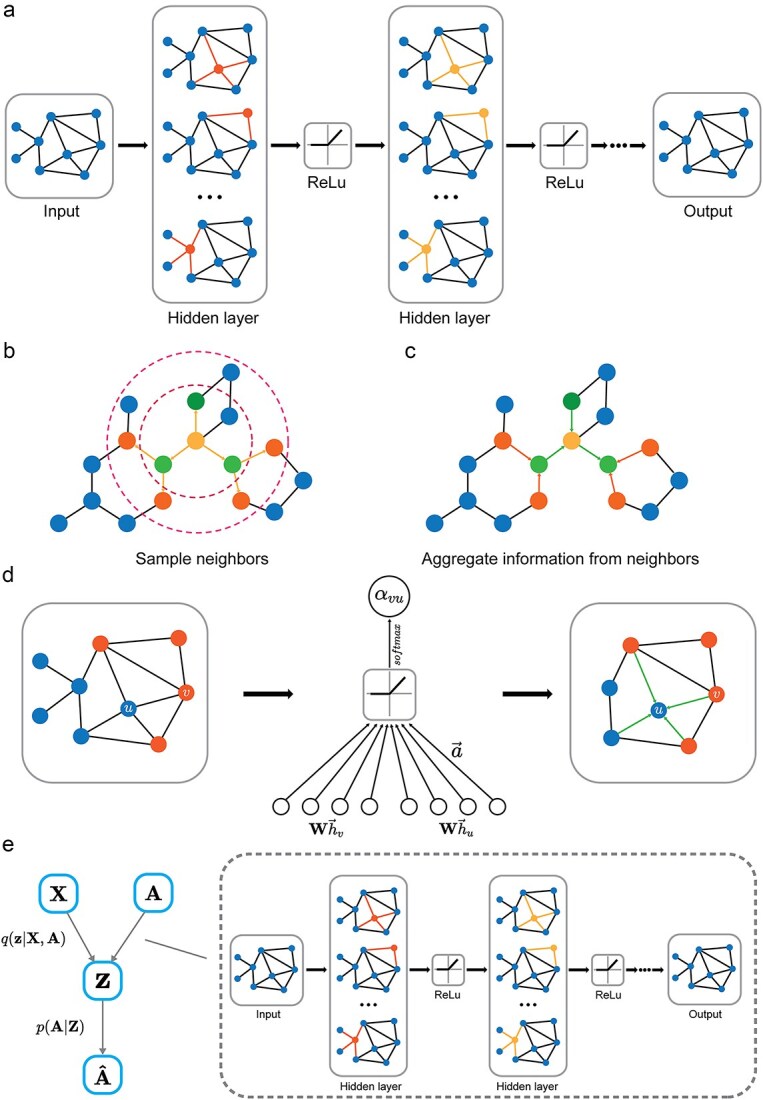
Overview of several GNN variants. (a) A typical pipeline of GCN, from input graph data and node features to feature extraction through convolutional layers, followed by non-linear activation and pooling for downstream tasks like node or graph classification. (b and c) An illustration of the information aggregation progress in GraphSAGE. In (b), the neighbor sampling process is shown, where a fixed-size subset of neighbors is selected for each node; In (c), these sampled neighbors’ features are aggregated using an appropriate aggregation function to update the node representations. (d) An illustration of GATs, where attention coefficients are computed to weight the importance of each neighbor’s features, allowing for adaptive aggregation based on the learned attention scores. (e) A pipeline of VGAEs, where graph data is encoded into latent space using a variational approach, followed by a decoder that reconstructs the graph structure, enabling unsupervised learning of graph representations.

GNNs have evolved into several key variants designed to tackle different challenges in graph learning [54–58, 73, 74]. GCNs extend traditional CNNs to graph-structured data by aggregating information from neighboring nodes (Fig. 2a). GraphSAGE improves scalability by using a sampling-based aggregation approach, making it more efficient for large graphs (Fig. 2b and2c). GATs introduce an attention mechanism that allows nodes to weigh the importance of their neighbors, enhancing the learning process (Fig. 2d). GTNs combine graph structures with transformer-based attention, enabling more flexible and dynamic learning of graph data. GAEs and VGAEs, based on autoencoder architectures, focus on unsupervised learning of graph representations, with VGAEs incorporating variational inference to model probabilistic graph structures (Fig. 2e). In the Supplementary Texts S2–S7, we provide a detailed illustration of these six key variants of GNNs, each of which introduces unique innovations to better capture graph structures and improve performance on a wide range of applications. There are also other GNN models, such as message passing neural networks (MPNNs; Supplementary Text S8). We also provided a brief discussion on the difference between GATs and GTNs in the Supplementary Text S9.

## Applications of graph neural network-based approaches for single-cell omics data analysis

**Figure 3:**
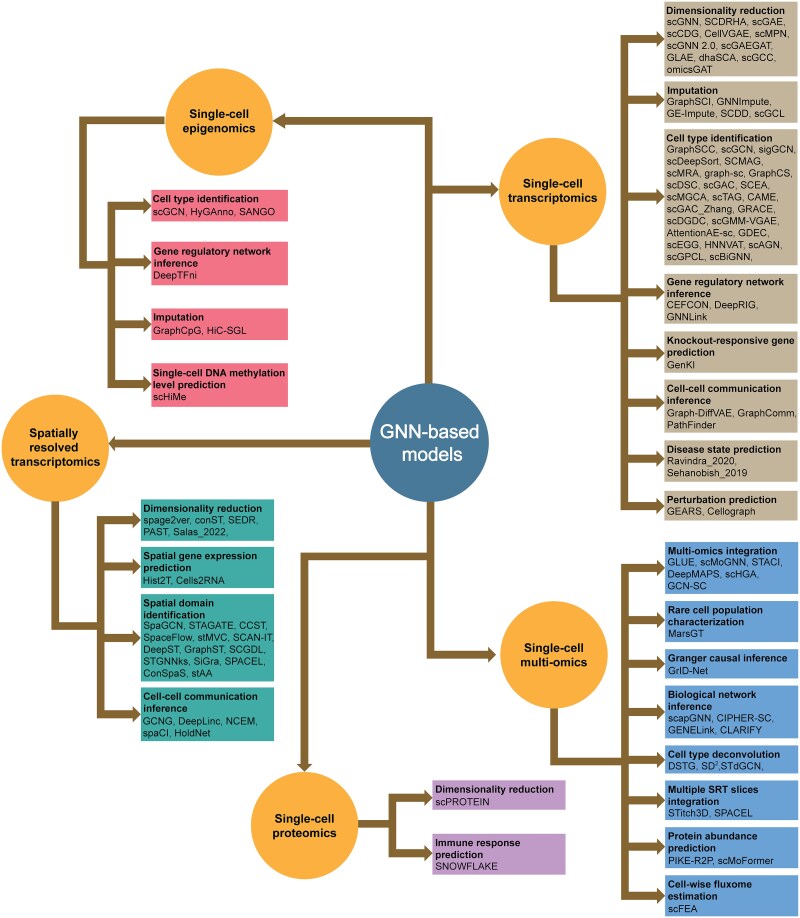
An overview of GNN-based models for various omics, categorized by the types of omics they process and the tasks they address.

Recent advancements in single-cell resolution technologies have revolutionized the profiling of chromatin accessibility, DNA methylation, and proteins, enhancing our understanding of the development, differentiation, aging, and various diseases and phenotypes at the genetic regulation and expression levels. Various derivatives of graph neural networks have been developed into hundreds of powerful bioinformatics computational methods for a diverse array of tasks in single-cell omics and single-cell multi-omics. In this section, we review the diverse applications of GNN-based computational methods in the analysis of single-cell omics data (Fig. 3). We also provided an overall introduction to the single-cell-processing pipeline for each application and the roles of GNNs in these pipelines in Supplementary Text S10.

### Graph neural networks for single-cell epigenomics

GNN-based methods have shown significant potential in analyzing single-cell epigenomics, providing a deeper understanding of the genome’s regulatory landscape. This section explores the applications of GNN-based methods in three fundamental areas of single-cell epigenomics: chromatin accessibility, DNA methylation, and chromosome conformation. These data types are often sparse, with each cell exhibiting distinct epigenetic marks that define its state. (i) Chromatin accessibility, which influences gene expression by determining the openness of chromatin regions, can be profiled at single-cell resolution using techniques like scATAC-seq, revealing key regulatory dynamics [75, 76]. (ii) DNA methylation, an important epigenetic modification involving methyl group addition to DNA, is essential for gene expression regulation and cell identity. scDNAm provides detailed information on methylation diversity and lineage relationships. (iii) Chromosome conformation refers to the 3D organization of the genome, which impacts gene regulation and genome stability. Single-cell Hi-C and related technologies allow researchers to capture spatial chromatin interactions, shedding light on the nuclear architecture. In the following sections, we will introduce various GNN-based methods and their applications in analyzing chromatin accessibility, DNA methylation, and chromosome conformation.

#### Chromatin accessibility

Cell type identification is a key task in the analysis of scCAS data, enabling the exploration of complex, cell-type-specific gene regulatory mechanisms and enhancing our understanding of cellular functions and their alterations in health and disease states [77, 78]. In terms of methodologies, cell type identification in scCAS data can be divided into two main approaches: transferring labels from well-annotated reference datasets from other omics and directly annotating cell types using well-labeled scCAS data as references. In the following sections, we discuss several GNN-based methods that exemplify these approaches.

For methods that transfer labels from well-annotated reference datasets from other omics to annotate scCAS data, Song *et al*. introduced scGCN [79], which effectively transfers labels from annotated scRNA-seq data to scCAS data in a semi-supervised manner using the scRNA-seq dataset as a reference. scGCN first establishes graph structures within the query dataset and between the reference and query data. It then utilizes a three-layer modified GCN [69] to annotate the cell type labels of the scCAS dataset using the labeled scRNA-seq dataset. The GCNs take the adjacency matrix of a hybrid graph composed of the above two graph structures and the concatenated feature matrices of the reference and query datasets. scGCN is trained by computing the loss function from the predicted results on the reference dataset against its known labels.

However, scGCN preprocesses scCAS data and converts peak features into gene activity, resulting in a training process that lacks informative peak-level features. HyGAnno [80] employs annotated scRNA-seq data as reference and trains a parallel VGAE model that incorporates both gene-level and peak-level features to transfer cell type labels to scCAS data. It demonstrated improved performance in cell type annotation across multiple datasets compared to scGCN.

In addition to methods that use other omics to annotate scCAS data, there are also methods like SANGO [81] that directly utilize well-labeled scCAS data to annotate cell types in new scCAS datasets. Initially, SANGO performs one-hot encoding of the DNA sequences corresponding to peaks in both the query scCAS dataset and the well-labeled reference scCAS dataset. It then uses a channel attention CNN to learn low-dimensional embeddings for cells from these encoded sequences. These learned embeddings, representing both the reference and query datasets, are subsequently fed into a GTN. The GTN is fine-tuned using the known cell type annotations from the reference dataset, which allows it to predict and annotate cell types in the query dataset. In this way, SANGO effectively performs cell type annotation for scCAS data.

#### Deoxyribonucleic acid methylation

DNA methylation is a key epigenetic modification involved in regulating gene expression and influencing development and disease. However, single-cell DNA methylation data often suffers from significant sparsity due to the limited DNA available from individual cells, leading to numerous missing values. To address this challenge, methylome imputation aims to infer these missing values, thereby providing a more complete understanding of methylation patterns and epigenetic regulation.

GraphCpG [82] addresses the challenge of methylome imputation by transforming the DNA methylation matrix into an undirected bipartite graph, where nodes represent either cells or loci, and edges depict the methylation state of a locus within a cell. The model first constructs this bipartite graph from the methylation data, with edges indicating known methylation states. Using a GCN-based model, GraphCpG learns embeddings for both cell and locus nodes by aggregating information from their neighbors, capturing relationships between similar cells and loci. These embeddings are then fed into a decoder to perform link prediction, effectively imputing missing methylation values by estimating the likelihood of connections between cells and loci. By leveraging the spatial and structural information inherent in the data, GraphCpG proves to be more effective than traditional statistical methods for methylome imputation.

#### Chromosome conformation

Chromosome conformation analysis at the single-cell level is challenging due to the high sparsity and complexity of the data, particularly with single-cell Hi-C (scHi-C), which aims to capture chromosomal interactions in individual cells. Compared to bulk Hi-C data, scHi-C data exhibits significantly higher sparsity, prompting the development of computational methods to impute missing chromosomal interactions.

To address the challenge of missing values, HiC-SGL [83] models scHi-C data as a set of graphs, with each cell represented by a graph whose nodes are chromosomal segments (bins) and edges represent interactions between these bins. HiC-SGL utilizes a cell-encoder to capture embeddings that reflect global features of the cell graph, and incorporates a transformer within the GNN to extract subgraph representations. The decoder predicts the existence probability of each edge within the cell graph. Furthermore, HiC-SGL has demonstrated the capacity of these cell embeddings to characterize cell heterogeneity through *k*-means clustering.

Beyond imputing chromosomal interactions, an important aspect of understanding chromosome conformation is to predict base-pair-specific DNA methylation levels, which are crucial for epigenetic regulation analysis. To address this problem, Zhu *et al.* proposed scHiMe [84], which predicts base-pair specific methylation levels in individual cells using scHi-C data and DNA nucleotide sequences. scHiMe incorporates the metacell strategy and applies a GTN-based model on the promoter-promoter spatial interaction network to predict DNA methylation levels.

In summary, GNN-based approaches have proven effective in single-cell epigenomics for tasks such as cell type identification, methylome imputation, and chromosomal interaction analysis. The graph structures of each method are summarized in Supplementary Table S1. Future work should aim to optimize these models for scalability and make their results more biologically interpretable. Additionally, GNNs could be explored for other tasks in single-cell epigenomics research, such as trajectory inference and integrating various single-cell omics data types, further broadening their impact on understanding complex cellular mechanisms.

### Graph neural networks for single-cell transcriptomics

Unachievable with bulk RNA sequencing techniques, scRNA-seq technologies provide granular insights into the transcriptome landscapes at the single-cell level across diverse species, samples, and tissues. scRNA-seq data has clearer semantic meaning, with each feature directly corresponding to the expression level of a gene. Compared to single-cell epigenomics data, scRNA-seq data is typically less sparse and has lower dimensionality, making it more manageable for computational models. The vast amount of single-cell transcriptomic data has spurred an explosive growth in computational tools designed to process and analyze these data (Table 2, Supplementary Text S11), owing to inherent challenges such as high noise levels, significant sparsity, and frequent dropouts [38].

**Table 2:** Details of GNN-based methods for single-cell transcriptomics.

Algorithm name	Model	Graph	Year	Task type	Reference
GraphSCI	GCNs	Gene–gene	2021	Imputation	[85]
GE-Impute	GNNs	Cell–cell	2022	Imputation	[86]
SCDD	GCNs	Cell–cell	2022	Imputation	[87]
GNNImpute	GATs	Cell–cell	2021	Imputation	[88]
scGCL	GCNs	Cell–cell	2023	Imputation	[89]
scGAEGAT	GAEs, GATs	Cell–cell	2022	Imputation, clustering	[90]
scGNN 2.0	GAEs, GATs	Cell–cell	2022	Imputation, clustering	[91]
scMPN	VGAEs	Cell–cell	2024	Imputation, clustering	[92]
scDGAE	GAEs, GCNs, GATs	Cell–cell	2023	Imputation, clustering	[93]
scGNN	GAEs, GCNs	Cell–cell	2021	Learn low-dimensional embedding	[63]
scGAE	GAEs, GATs	Cell–cell	2021	Learn low-dimensional embedding	[94]
GLAE	GAEs, GCNs	Cell–cell	2022	Learn low-dimensional embedding	[95]
scCDG	GAEs, GCNs	Cell–cell	2021	Learn low-dimensional embedding	[96]
scGCC	GATs	Cell–cell	2023	Learn low-dimensional embedding	[97]
SCDRHA	GAEs, GATs	Cell–cell	2021	Learn low-dimensional embedding	[98]
omicsGAT	GATs	Cell–cell	2022	Learn low-dimensional embedding	[99]
CellVGAE	VGAEs, GATs	Cell–cell	2021	Learn low-dimensional embedding	[100]
dhaSCA	GCNs	Cell–cell	2023	Learn low-dimensional embedding	[101]
scDGDC	GCNs	Cell–cell	2023	Learn low-dimensional embedding	[102]
AttentionAE-sc	GAEs, GCNs	Cell–cell	2023	Learn low-dimensional embedding	[103]
scDSC	GNNs	Cell–cell	2022	Learn low-dimensional embedding	[104]
SCEA	GAEs	Cell–cell	2023	Learn low-dimensional embedding	[105]
scTAG	GAEs	Cell–cell	2022	Learn low-dimensional embedding	[106]
scGCN	GCNs	Cell–cell	2021	Label transfer	[79]
SCMAG	GCNs	Cell–cell	2021	Cell type annotation	[107]
GraphCS	GNNs	Cell–cell	2022	Cell type annotation	[108]
graph-sc	GAEs, GCNs	Gene-cell	2021	Cell type annotation	[109]
HNNVAT	GCNs	Cell–cell	2023	Cell type annotation	[110]
sigGCN	GCNs	Gene–gene	2021	Cell type annotation	[111]
scBiGNN	GATs	Gene–gene and cell–cell	2023	Cell type annotation	[112]
CAME	GCNs	Cell–cell, cell-gene, and gene–gene	2022	Cell type annotation	[113]
scDeepSort	GraphSAGE	Cell-gene	2021	Cell type annotation	[114]
scMRA	GCNs	Prototype-prototype, prototype-cell, and cell–cell	2021	Cell type annotation	[115]
scAGN	GATs	Cell–cell	2023	Cell type annotation	[116]
GraphSCC	GCNs	Cell–cell	2021	Clustering	[117]
scGPCL	GNNs	Cell-gene	2023	Clustering	[118]
scMGCA	GAEs	Cell–cell	2023	Clustering	[119]
scGAC	GAEs, GATs	Cell–cell	2022	Clustering	[120]
scEGG	GAEs, GATs	Cell–cell	2024	Clustering	[121]
GDEC	GCNs	Gene–gene	2023	Clustering	[122]
GRACE	GAEs	Cell–cell	2023	Clustering	[123]
scASGC	GCNs	Cell–cell	2023	Clustering	[124]
scGMM-VGAE	VGAEs, GCNs	Cell–cell	2023	Clustering	[125]
CEFCON	GATs	Gene–gene	2023	Gene regulatory network inference	[126]
DeepRIG	GAEs, GCNs	Gene–gene	2023	Gene regulatory network inference	[127]
GNNLink	GCNs	Gene–gene	2023	Gene regulatory network inference	[128]
GenKI	VGAEs	Gene–gene	2023	KO-responsive gene identification	[129]
PathFinder	GTNs	Gene–gene	2024	Cell–cell communication inference	[47]
Graph-DiffVAE	VGAEs, GCNs	Cell–cell	2020	Cell–cell communication inference	[131]
GraphComm	GATs	Protein–protein	2023	Cell–cell communication inference	[132]
Ravindra_2020	GATs	Cell–cell	2020	Disease state prediction	[60]
Sehanobish_2021	GATs	Cell–cell	2021	Disease state prediction	[192]
GEARS	GNNs	Gene–gene	2023	Single-cell perturbation prediction	[193]
Cellograph	GCNs	Cell–cell	2024	Single-cell perturbation prediction	[194]

#### Imputation

Generally, cells with identical functions often exhibit similar features, which inspired us to recover dropouts in gene expression profiles by leveraging similar cells. GNN-based methods for scRNA-seq data imputation can be categorized based on their different strategies for constructing cellular relationships and enhancing imputation through graph learning.

One common approach is to leverage cellular similarity metrics to construct a graph that represents relationships between cells, such as using *k*-nearest neighbors (*k*NN), Pearson correlation, or Spearman correlation to create similarity graphs [85–87, 130]. For example, GraphSCI [85] leverages the Pearson correlation coefficient to construct a gene co-expression graph from raw gene expression data. It then utilizes GCN to encode expression levels and reconstruct the co-expression network. Concurrently, an autoencoder is employed to sample the encoded embeddings, thus imputing the gene expression matrix.

Another approach of methods enhances imputation performance by incorporating dynamic weighting of cellular relationships. GNNImpute [88] incorporates a graph attention mechanism to dynamically assign different weights to similar cells on a *k*NN graph, which is constructed based on principal component analysis of the preprocessed gene expression matrix.

#### Dimensionality reduction

Dimensionality reduction is a critical task in single-cell transcriptomics data analysis, as scRNA-seq data is often high-dimensional and noisy, making downstream analyses challenging. GNN-based methods have been applied to dimensionality reduction, offering an effective way to learn low-dimensional representations of cells. To better organize the numerous GNN-based models for dimensionality reduction, we classify them based on their core architecture and methodology.

GAE-based methods employ autoencoders with graph structures to learn low-dimensional embeddings that capture the relationships between cells. These methods typically start by constructing a cell graph, followed by utilizing an autoencoder framework to derive meaningful representations [63, 94–96]. scGNN [63] is a representative GAE-based model that employs four stacked autoencoders for scRNA-seq analysis. It begins by learning feature embeddings, constructs a cell–cell *k*NN graph, prunes it, and applies a GAE to derive low-dimensional representations. These representations are then used for clustering, followed by iterative gene expression reconstruction until clustering stabilizes. Finally, an imputation autoencoder reconstructs the gene expression matrix.

#### Cell type identification

Identifying cell types from scRNA-seq data is a crucial application of GNNs, and a diverse set of methods has been developed, each taking a different approach to leveraging graph structures and neural network architectures. These methods can be organized into distinct categories based on how they define cell relationships, the types of data they incorporate, and their underlying learning strategies.

The success of GNN-based cell type identification relies heavily on how the graph is constructed to represent relationships within scRNA-seq data. Different approaches have been developed based on cell similarity, gene–gene interactions, or combining heterogeneous data. The most common strategy for GNN-based cell type identification is constructing a cell similarity graph, where nodes represent cells and edges capture relationships based on similarity metrics [107, 108, 117]. For instance, GraphSCC [117] leverages a denoising autoencoder along with a GCN applied to a *k*NN-based cell graph, integrating dual self-supervised learning to enhance the quality of cell clustering.

#### Cell–cell communication inference

Cell-to-cell communication is crucial for understanding interactions in multi-cellular systems, coordination of biological processes, and changes in different conditions like development or disease. Below, we introduce three GNN-based methods that have been developed to infer cell–cell interactions.

Graph-DiffVAE [131] begins by constructing an undirected and unweighted cell–cell graph represented by a binary adjacency matrix based on Pearson correlation coefficients between cells. Subsequently, this adjacency matrix, combined with cell features is fed into a VGAE-based model to predict relationships between cells of various types, aiding in the understanding of cell differentiation. Moreover, GraphComm [132] combines an annotated ligand-receptor database and utilizes a GAT to predict cell–cell communication interactions for scRNA-seq data. PathFinder [47] employs a divide-and-conquer approach to tackle this complexity. By partitioning multi-cellular intra- and inter-cellular signaling networks into smaller, more manageable signaling paths, PathFinder uses a GTN-based framework to deduce the interactions both within and between cells.

### Graph neural networks for spatially resolved transcriptomics

Previously, array-based ST technologies could only measure gene expression at the spot level, which allowed for the estimation of cell-type compositions in each spot but could not distinguish individual cell types within a spot. Recent technology advances in SRT have facilitated the capture of cellular spatial relative localization histological imaging, and gene expression profiles at single-cell and even sub-cellular resolutions, providing deeper insights into complex disease mechanisms [67, 133, 134]. This modality provides gene expression profiles along with the spatial coordinates of cells, which requires GNN-based models to process two types of data simultaneously and learn the correspondence between spatial structure and gene expression. Consequently, the graph structure must encode not only gene expression similarity but also spatial neighborhood information [135]. In the following sections, we will explore how GNN-based models can be applied to SRT data to reveal intricate spatial interactions and enhance biological insights (Table 3, Supplementary Text S12).

**Table 3:** Details of GNN-based methods for SRT.

Algorithm name	Model	Graph	Year	Task type	Reference
Hist2ST	GraphSAGE	Spot-spot	2022	Spatial gene expression prediction	[136]
Cells2RNA	GATs	Cell–cell	2023	Spatial gene expression prediction	[137]
spage2vec	GraphSAGE	Spot-spot	2020	Learn low-dimensional embedding	[138]
Salas_2022	GraphSAGE	Spot-spot	2022	Learn low-dimensional embedding	[139]
conST	VGAEs	Cell–cell or spot-spot	2022	Learn low-dimensional embedding	[140]
PAST	VGAEs	Spot-spot	2023	Learn low-dimensional embedding	[141]
SEDR	VGAEs, GCNs	Cell–cell or spot-spot	2024	Learn low-dimensional embedding	[142]
CCST	GCNs	Cell–cell or spot-spot	2022	Clustering	[143]
SCGDL	GCNs	Spot-spot	2023	Clustering	[144]
STAGATE	GAEs, GATs	Cell–cell or spot-spot	2022	Spatial domain identification	[145]
stMVC	GATs	Cell–cell or spot-spot	2022	Spatial domain identification	[146]
GraphST	GCNs	Spot-spot	2023	Spatial domain identification	[147]
SpaGCN	GCNs	Cell–cell or spot-spot	2021	Spatial domain identification	[133]
SpaceFlow	GCNs	Cell–cell or spot-spot	2022	Spatial domain identification	[148]
SCAN-IT	GCNs	Cell–cell or spot-spot	2022	Spatial domain identification	[149]
DeepST	VGAEs	Cell–cell or spot-spot	2022	Spatial domain identification	[150]
SiGra	GTNs	Cell–cell or spot-spot	2023	Spatial domain identification	[151]
SPACEL	GCNs	Cell–cell or spot-spot	2023	Spatial domain identification	[152]
STGNNks	GCNs	Spot-spot	2023	Spatial domain identification	[153]
ConSpaS	GAEs	Spot-spot	2023	Spatial domain identification	[154]
stAA	VGAEs	Cell–cell or spot-spot	2024	Spatial domain identification	[155]
GCNG	GCNs	Cell–cell	2020	Ligand-receptor interaction inference	[156]
spaCI	GATs	Cell–cell	2022	Ligand-receptor interaction inference	[157]
DeepLinc	VGAEs, GCNs	Cell–cell	2022	Cell–cell communication inference	[41]
HoldNet	GATs, GCNs	Cell–cell	2023	Cell–cell communication inference	[158]
NCEM	GCNs, GATs	Cell–cell	2022	Cell–cell communication inference	[159]

#### Spatial gene expression prediction

Spatial gene expression prediction aims to estimate gene expression levels from spatial information, such as histological images, enabling researchers to bridge the gap between visual tissue morphology and molecular data. This task is particularly valuable for enhancing our understanding of the spatial organization.

Hist2ST [136] is a method designed to predict gene expression directly from histology images. Hist2ST begins by segmenting the histology image into patches and extracting 2D vision features from these patches. Subsequently, Hist2ST learns spot features with global spatial dependencies through a transformer module and constructs a spot *k*NN graph based on Euclidean distance, which is then fed into a GraphSAGE-based GNN to incorporate local spatial dependencies. Additionally, Hist2ST utilizes a ZINB model to capture the features learned and output the predicted gene-by-spot expression matrix.

#### Dimensionality reduction

In SRT analysis, dimensionality reduction remains a crucial step. By projecting the data into a lower-dimensional space, researchers can better capture spatial heterogeneity and facilitate downstream analyses that are specific to spatial data, such as identifying spatial domains, inferring spatial patterns, and correcting batch effects. Below, we present several GNN-based methods that employ dimensionality reduction for ST, highlighting their different strategies and advantages.

One common approach involves constructing cell or spot graphs based solely on spatial coordinates [138, 139]. spage2vec [138] employs GraphSAGE to encode low-dimensional representations from a *k*NN graph constructed using spatial information. The learned representations characterize spatial transcriptomic heterogeneity and facilitate spatial domain identification through the Leiden clustering algorithm.

#### Spatial domain identification

Spatial domain identification in SRT aims to delineate spatially coherent domains within tissues based on gene expression, spatial localization, and other available data modalities [160–162]. By identifying these spatial domains, researchers can better understand how cellular environments influence gene expression and how different tissue regions contribute to overall function or pathology. Below, we introduce several GNN-based methods that have been developed for spatial domain identification, categorized by their graph construction techniques, learning paradigms, and data integration strategies [133, 145–152].

The most basic approach for identifying spatial domains involves constructing graphs that represent spatial dependencies among spots or cells. SpaGCN [133] constructs an undirected weighted graph that represents spatial dependencies from SRT data. Each node represents a spot or cell and the edge weights between two nodes are determined by their level of relatedness. SpaGCN aggregates spatial localization, histology information, and gene expression via a GCN, employs iterative clustering to identify spatial domains, and detects spatially variable genes enriched in each domain.

#### Cell–cell communication inference

Cell–cell communications can be categorized into intracellular and intercellular interactions [41, 156–158]. GCNG [156], a supervised GCN-based approach, is specifically designed for delineating intercellular gene interactions, particularly ligand-receptor communication, from single-cell SRT data. Taking the spatial cell location and gene expression as input, GCNG also constructs a cell–cell graph by computing the Euclidean distance between cells utilizing their spatial coordinates. Instead of using *k*NN, GCNG selects neighboring cells according to a predefined threshold of distance to construct the cell neighborhood graph.

### Graph neural networks for single-cell proteomics

Proteins are the direct executors of cellular activities. Despite insights from scRNA-seq data about gene expression, the correlation between mRNA and protein levels often lacks consistency [163], which may lead to inaccuracies when inferring protein abundance solely based on scRNA-seq profiles. Recent advancements in single-cell proteomics based on mass spectrometry and next-generation sequencing methods have significantly enhanced our understanding of cellular heterogeneity, cell function, and disease mechanisms [163–166], yielding thrilling insights. Proteomic data typically has clear semantic meaning, as the features represent actual protein quantities. However, due to current technological constraints, single-cell proteomics data often has lower dimensionality, with only dozens to hundreds of protein features measurable per cell, requiring GNNs must be adapted to handle these issues.

scPROTEIN [167] treats each cell in the single-cell proteomic data abundance matrix as a node, calculates the Pearson correlation coefficient between abundance features to establish edges, and thus constructs an undirected and unweighted graph. scPROTEIN learns cell embeddings from cell-by-protein matrix based on GCN [54], and has demonstrated the successful application of these embeddings in various downstream analyses including cell clustering, classification, and batch correction.

SNOWFLAKE [168] integrates single-cell proteomics, morphological, and structural data, modeling single-cell neighboring information from imaging data as single-cell spatial graphs. Based on a GNN-based framework, it forecasts immune reactions and assesses the distinctiveness of tissue microenvironments. The graph structures for each method are presented in Supplementary Table S2.

Proteins do not function in isolation; instead, they interact within complex networks, engaging in signaling pathways, forming protein complexes, and affecting cellular activities in tandem. Therefore, these protein–protein interactions are naturally modeled as networks, providing an ideal setting for leveraging GNN-based frameworks to capture such intricate relationships. Future applications of GNN-based models could include the development of integrative frameworks to combine proteomic data with other omics, enhancing our understanding of protein-level regulation, or employing GNNs to predict protein function and interaction in different cell states, which will be introduced in the next section. The potential to advance single-cell proteomic research using GNNs remains vast, warranting further exploration and innovation.

### Graph neural networks for single-cell multi-omics

Single-cell multi-omics methods allow for the measurement of different molecular aspects from individual cells, including the genome, epigenome, transcriptome, proteome, or metabolome. While this modality combines information from multiple data types, it also introduces complexities due to the heterogeneity of data types. Each omics layer has its own feature space and scale, making integration challenging. Therefore, GNNs applied to multi-omics data must be designed to integrate heterogeneous data types into a unified representation. The integrated analysis of these layers has revealed complex interactions across multi-omics, providing invaluable insights into cellular biology [66, 75] (Table 4, Supplementary Text S13).

**Table 4:** Details of GNN-based methods for single-cell multi-omics.

Algorithm name	Model	Graph	Year	Task type	Reference
GLUE	VGAEs	Feature-feature	2022	Integration	[169]
GCN-SC	GCNs	Cell–cell	2023	Integration	[170]
STitch3D	GATs	Spot-spot	2023	Multiple SRT slices integration	[171]
scMoGNN	GCNs	Cell-feature	2022	Modality prediction, modality matching	[172]
STACI	VGAEs	Cell–cell	2022	Learn joint low-dimensional embedding	[173]
scHGA	GNNs	Cell-gene-loci	2023	Learn joint low-dimensional embedding	[174]
MarsGT	GTNs	Cell-gene and cell-peak	2024	Rare cell population identification	[175]
DeepMAPs	GAEs, GTNs	Cell-gene	2023	Integration and cell-type-specific biological network inference	[64]
GrID-Net	GNNs	Cell–cell	2022	Granger causal inference	[176]
scapGNN	VGAEs	Cell–cell or gene–gene	2023	Active pathway inference and gene module identification	[177]
CIPHER-SC	GCNs	Disease-gene	2020	Disease-gene association inference	[178]
GENELink	GATs	Gene–gene	2022	Gene regulatory network inference	[179]
CLARIFY	GAEs	Cell–cell and gene–gene	2023	Cell–cell interaction inference	[180]
$\mathrm{S}{\mathrm{D}}^2$	GCNs	Spot-spot	2022	ST deconvolution	[181]
DSTG	GCNs	Spot-spot	2021	Cell type deconvolution	[182]
STdGCN	GCNs	Spot-spot	2023	Cell type deconvolution	[183]
SPACEL	GCNs	Cell–cell or spot-spot	2023	Spatial domain identification	[152]
PIKE-R2P	GNNs	Protein–protein	2021	Protein abundance prediction	[184]
scMoFormer	GTNs	Cell-gene, gene-protein, gene–gene, and protein–protein	2023	Protein abundance prediction	[185]
scFEA	GNNs	Metabolite-metabolic module	2021	Cell-wise fluxome prediction	[186]

#### Multi-omics integration

Single-cell multi-omics integration methods align cell states across various omics layers to provide a unified view of cellular heterogeneity. Recent advancements in GNN-based models for multi-omics integration focus on learning unified cell representations by constructing heterogeneous or multi-relational graphs that combine diverse single-cell data types, such as transcriptomics, epigenomics, and spatial data.

A common approach for multi-omics integration is aligning different omics modalities through advanced embedding strategies [64, 169, 172]. For example, GLUE [169] utilizes a VGAE-based model to integrate single-cell transcriptomics, and single-cell epigenomics (scCAS and scDNAm) data. GLUE initially applies omics-specific VAEs to yield low-dimensional cell representations for each of the three omics. To ensure that the learned cell embeddings from different omics layers have uniform semantics, GLUE incorporates a guidance graph based on prior knowledge of regulatory interactions, with nodes representing features from each omics layer and edges representing the sign and credibility of the regulatory interaction. Finally, GLUE employs an omics discriminator to align the cell embeddings across different omics, thus integrating the data from all three omics.

#### Rare cell population characterization

Rare cell populations play a critical role in complex disease processes, such as cancer progression and immune response. The ability to accurately characterize these rare populations provides valuable insights into disease mechanisms and potential therapeutic targets. MarsGT [175] is a notable method that addresses the challenge of rare cell population characterization by integrating paired scRNA-seq and scATAC-seq data into a heterogeneous graph structure. In this graph, nodes represent cells, genes, and peaks, while edges capture the interactions between cells and genes or cells and peaks. By modeling these relationships, MarsGT uses a probability-based heterogeneous graph transformer framework to discern cell clusters, focusing especially on rare cell populations that may be underrepresented in typical clustering approaches. MarsGT further predicts cluster-specific peak-gene interactions and infers enhancer gene regulatory networks (eGRNs), which play a vital role in understanding gene regulation within rare cell populations. This comprehensive integration of multiple data types and advanced modeling of interactions allows MarsGT to effectively identify these elusive yet biologically significant cell clusters.

#### Biological network inference

Biological networks are key to unraveling cellular heterogeneity and interpreting cellular mechanisms. Below, we discuss several GNN-based approaches for biological network inference, emphasizing their unique contributions and methods for mitigating limitations in previous techniques.

Han *et al.* proposed scapGNN [177] to infer gene-cell, gene–gene, and cell–cell association networks. scapGNN aims to infer gene-cell, gene–gene, and cell–cell association networks by leveraging both scRNA-seq and scCAS data. It starts by converting single-cell data into pathway activity score matrices to characterize cellular heterogeneity, allowing for a detailed examination of cellular states. Starting from gene expression of scRNA-seq data or gene activity matrices from scCAS data, scapGNN learns low-dimensional embeddings of cells and genes using a deep neural network autoencoder. It constructs gene correlation and cell correlation networks separately from the gene-cell matrix using Pearson correlation coefficients, then learns gene–gene and cell–cell association networks with VGAEs, incorporating gene and cell embeddings separately. Using the random walk with restart algorithm, it infers a weighted gene-cell association network and generates a pathway-by-cell activity matrix, which aids in clustering and identifying gene modules that are crucial for depicting specific cell phenotypes. Finally, scapGNN integrates inferred networks from both two omics using Brown’s method [187].

#### Cell type deconvolution

Cell type deconvolution is an essential task in ST, aiming to estimate the proportions of different cell types present within each spatial spot. Below, we describe several GNN-based methods that utilize multi-omics data to tackle this task effectively.

DSTG [182] utilizes a GNN to consider the previously overlooked topological information between spots to deconvolute SRT data. It constructs pseudo-ST data using annotated scRNA-seq data, subsequently applying canonical correlation analysis to conduct dimensionality reduction on both pseudo and real SRT data. DSTG then connects pseudo and real SRT data into a spot neighborhood graph, linking spots considered as mutual *k*-nearest neighbors. It inputs the link graph along with variable gene expression matrices from both pseudo and real SRT data into a GCN to predict cell type proportions at each spot through semi-supervised learning by calculating cross-entropy between the actual cell proportions and the predicted compositions of the pseudo-ST data.

**Figure 4:**
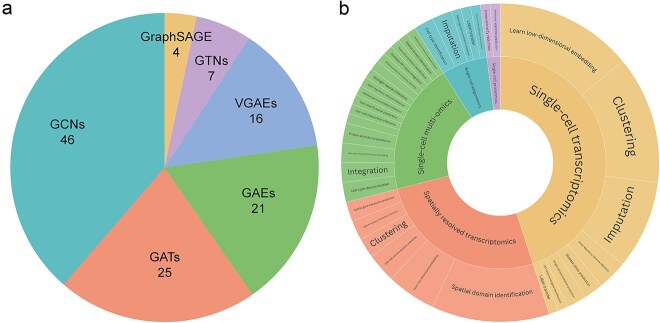
Distribution and growth of GNN-based models. (a) The pie chart shows the proportion of six different GNN variants in the review. (b) The sunburst chart illustrates the successful application of GNNbased models across various tasks in multiple single-cell omics data analysis.

In summary, GNN-based methods have emerged as powerful tools for analyzing single-cell omics data, significantly advancing our understanding of genetic regulation and expression. As shown in Fig. 4, GNN-based frameworks, such as GCNs, GATs, and GAEs, have been widely utilized in the analysis of single-cell omics data. Additionally, a variety of GNN-based models have been successfully developed to tackle a broad spectrum of tasks in single-cell omics, including cell type identification, dimensionality reduction, and biological network inference. These advancements hold great promise for unlocking deeper insights into complex biological systems, supporting the development of more precise diagnostic and therapeutic strategies. We have summarized the principal framework for applying GNNs to single-cell omics in Supplementary Text S14 for insightful guidance. We also collected a total of 77 GNN-based methods commonly used publicly available datasets in Supplementary Tables S3–S7 to provide readers with practical resources for their own studies according to the type of omics. We explain the collection of these data in Supplementary Text S15.

## Discussion: challenges and opportunities

Throughout over a hundred computational methods for various single-cell omics data reviewed in this survey, many of them model the single-cell resolution data as cell–cell or gene–gene graphs to characterize the data via graph structures, which are then fed into subsequent GNN-based networks. When constructing these graphs, it is widely adopted to calculate the Euclidean distance or assess other similarity metrics between cells and then establish the cell neighborhood graphs via *k*NN with varying values of *k*, thresholds of similarity metrics, or other criteria to select neighbors. This provides a solid reference and opens potential avenues for further refinement.

Incorporating external information into GNN-based models has become an essential strategy to enhance biological relevance and interpretability. For example, incorporating protein–protein interaction networks, gene regulatory networks, and prior biological knowledge of ligand-receptor interactions provides valuable context, enabling more accurate modeling of complex biological systems. These external sources help GNNs capture functional relationships that might not be evident from the data alone, such as regulatory interactions or intercellular communication pathways.

GNNs, by their nature, have high computational demands, especially as the number of nodes and edges increases, which presents a significant challenge in terms of scalability and efficiency. For example, for single-cell transcriptomics, where each cell or gene is treated as a node, the sheer scale of data often leads to excessive memory requirements and prolonged training times. This makes the selection of input features a challenging and critical step, as processing the full transcriptome could quickly become impractical for most models. To mitigate these challenges, feature selection techniques are often employed to reduce the dimensionality of the data. Selecting only highly variable genes or highly expressed genes can significantly cut down the model size, making GNNs more tractable while retaining essential biological information. However, this approach also presents a trade-off: reducing the data to manage computational feasibility may result in the loss of information critical for capturing nuanced biological behaviors [4, 188]. The inherent scalability limitations of GNNs necessitate careful optimization of these trade-offs to ensure that computational feasibility is maintained without compromising model performance or biological insight.

Another challenge of GNNs is their tendency to aggregate node information based primarily on local neighborhoods, making it challenging to capture global structural information, which is essential for tasks requiring long-range dependencies, such as inferring gene regulatory networks or understanding cellular differentiation. A common solution to this issue is increasing the depth of the network, allowing for the aggregation of information over larger node neighborhoods. However, this solution introduces more challenges. On the one hand, as the network depth increases, the computational complexity grows exponentially, making deep GNNs difficult to train on large-scale single-cell data. On the other hand, increasing the number of layers exacerbates the issue of over-smoothing, where node representations become overly similar, leading to a loss of discriminative ability. In single-cell transcriptomics, for instance, over-smoothing could result in different cell types or states being indistinguishable, thereby hindering the model’s ability to accurately annotate cells or predict their responses. Therefore, capturing global structural information and avoiding over-smoothing are in tension, making this another trade-off that requires careful optimization to ensure the model can effectively capture global dependencies without compromising the ability to distinguish between important biological features.

With the emergence of foundation models [189–191] pre-trained on large single-cell transcriptomics datasets, there is a growing opportunity for GNN-based models to leverage these models’ generalization capabilities. These pre-trained GNNs, having learned from complex biological data, capture patterns in gene expression and regulatory mechanisms across diverse conditions, offering a strong starting point for downstream tasks like cell type classification or gene network inference. Fine-tuning these pre-trained GNN models on smaller, task-specific datasets can significantly boost performance, especially in scenarios with limited labeled data.

Furthermore, with advancements in various technologies, such as high-resolution imaging and single-cell proteomics, data with subcellular-level resolution has become increasingly available. This creates a significant opportunity for GNN-based frameworks to manage and analyze such detailed datasets. Moreover, the integration of subcellular data with other types of omics data, such as transcriptomics and epigenomics, could enable GNNs to develop more comprehensive models of cellular behavior. This approach could lead to breakthroughs in understanding complex diseases and developing personalized medicine strategies, paving the way for more innovative and accurate single-cell data analyses.

## Conclusion

Our survey begins by introducing the basic structure of GNNs and their six primary variants, followed by a detailed exploration of how GNN-based models are applied across different single-cell omics, highlighting specific implementation methods and approaches. Our review has documented 107 successful applications of GNN-based frameworks in a wide array of tasks involving single-cell epigenomics, transcriptomics, ST, proteomics, and multi-omics. These methods have demonstrated significant utility in understanding cellular heterogeneity, spatial domain identification, biological network inference, cell–cell communication, Granger causal inference, cell type deconvolution, multiple SRT slices integration, and the integration of multi-omics data. Besides, in each omics section, we also comprehensively summarize the publicly available single-cell data of the corresponding omics that are widely used by the GNN-based approaches reviewed in that section. We anticipate that the extensive collection of datasets we have compiled will provide readers with valuable resources for their own research. Additionally, we have highlighted several key considerations in graph construction, feature selection, integration of external information, and the use of foundation models. These findings suggest new potential directions for future experimentation, including enhancing biological interpretability and improving computational scalability. We anticipate that this survey will serve as a resource for researchers aiming to deepen their understanding of deep learning applications in single-cell analysis, providing both practical guidelines for adapting GNNs to single-cell omics and valuable insights for the broader development of computational methods in the field.

**Key Points**

- This review introduces six key variants of Graph neural networks (GNNs)—GCNs, GraphSAGE, GATs, GTNs, GAEs, and VGAEs—and their roles in single-cell omics analysis.
- This study systematically reviews 107 applications and commonly used publicly available benchmark datasets of GNN-based models in single-cell epigenomics, transcriptomics, spatial transcriptomics, proteomics, and multi-omics for various computational tasks.
- We discuss various graph construction strategies including cell–cell, gene–gene, and heterogeneous graphs, tailored to address the complexity of biological relationships.
- We highlight the successful application of GNN-based frameworks and outline potential avenues for future research in applying GNNs to single-cell omics.

## Supplementary Material

supplementary_bbaf109

## Data Availability

No data was used in this study.
