# Supplementary material for: Graph neural networks for single-cell omics data: a review of approaches and applications
Source: Brief Bioinform. 2025 Mar 17;26(2):bbaf109. doi: 10.1093/bib/bbaf109 (PMC11911123; doi:10.1093/bib/bbaf109)
Supplement: supplementary_bbaf109 [file supplementary_bbaf109.docx]

**Supplementary Information for**

**Graph neural networks for single-cell omics data: a review of approaches and applications**

Sijie Li^1,†^, Heyang Hua^1,†^ and Shengquan Chen^1,^*

^1^ School of Mathematical Sciences and LPMC, Nankai University, Tianjin 300071, China

^*^ Corresponding author: chenshengquan@nankai.edu.cn

^†^ These authors contributed equally: Sijie Li and Heyang Hua

**Contents**

[Supplementary Texts 3](#_Toc191324868)

[Text S1. An introduction of the performance comparison of GNN-based approaches against traditional methods 3](#_Toc191324869)

[Text S2. Graph convolutional networks (GCNs) 5](#_Toc191324870)

[Text S3. GraphSAGE 6](#_Toc191324871)

[Text S4. Graph attention networks (GATs) 7](#_Toc191324872)

[Text S5. Graph transformer networks (GTNs) 8](#_Toc191324873)

[Text S6. Graph autoencoders (GAEs) 9](#_Toc191324874)

[Text S7. Variational graph autoencoders (VGAEs) 10](#_Toc191324875)

[Text S8. Message passing neural networks (MPNNs) 11](#_Toc191324876)

[Text S9. Differences between GATs and GTNs 12](#_Toc191324877)

[Text S10. An overall introduction to the single-cell-processing pipeline for each application and the roles of GNNs in these pipelines 13](#_Toc191324878)

[Text S11. GNNs for single-cell transcriptomics 15](#_Toc191324879)

[Text S12. GNNs for spatially resolved transcriptomics 18](#_Toc191324880)

[Text S13. GNNs for single-cell multi-omics 19](#_Toc191324881)

[Text S14. The principle framework on how to apply GNNs to single-cell omics 21](#_Toc191324882)

[Text S15. Explanation on the collection of commonly used single-cell datasets in GNN-based approaches 22](#_Toc191324883)

[Supplementary Tables 23](#_Toc191324884)

[Table S1. Details of GNN-based methods for single-cell epigenomics. 23](#_Toc191324885)

[Table S2. Details of GNN-based methods for single-cell proteomics. 24](#_Toc191324886)

[Table S3. Publicly available single-cell epigenomics datasets commonly used by 107 GNN-based methods in this review. 25](#_Toc191324887)

[Table S4. Publicly available single-cell transcriptomics datasets commonly used by 107 GNN-based methods in this review. 26](#_Toc191324888)

[Table S5. Publicly available single-cell spatially resolved transcriptomics datasets commonly used by 107 GNN-based methods in this review. 27](#_Toc191324889)

[Table S6. Publicly available single-cell proteomics datasets commonly used by 107 GNN-based methods in this review. 28](#_Toc191324890)

[Table S7. Publicly available single-cell multi-omics datasets commonly used by 107 GNN-based methods in this review. 29](#_Toc191324891)

[References 30](#_Toc191324892)

**Supplementary Texts**

**Text S1. An introduction of the performance comparison of GNN-based approaches against traditional methods**

In this section, we have systematically summarized the 107 studies included in our review, many of which include benchmark comparisons with traditional methods. We extracted relevant benchmarking results from these articles to provide insights into how GNN-based approaches perform relative to traditional methods. We categorize traditional methods into two distinct groups: (1) widely adopted task-specific approaches designed for single-cell data analysis, and (2) general-purpose traditional machine learning techniques. A detailed elaboration follows below.

Taking extracellular ligand-receptor interactions, a critical component of cell-cell communication, as an example, GCNG [1] a supervised GCN-based approach, is specifically designed for delineating intercellular gene interactions, particularly ligand-receptor communication, from single-cell SRT data. Taking the spatial cell location and gene expression as input, GCNG also constructs a cell-cell graph through computing the Euclidean distance between cells utilizing their spatial coordinates. Instead of using *k*-nearest neighbors (*k*NN), GCNG selects neighboring cells according to a predefined threshold of distance to construct the cell neighborhood graph. To evaluate the capability of GCNG in predicting extracellular ligand-receptor interactions, the authors compared its performance against three traditional methods on two SRT datasets from seqFISH+ and MERFISH. The three traditional methods include Giotto [2], which utilizes spatial information to predict genes involved in extracellular gene interactions. The second method calculates the spatial Pearson’s correlation (PC) between ligands and receptors in neighboring cells, referred to as spatial PC. The third method, named single cell PC, does not utilize spatial information and is based on Pearson’s correlation between the expression of ligands and receptors within each cell. As illustrated in Figs. 2e and 2f of the GCNG article, GCNG demonstrated superior performance in both area under the receiver operating characteristic curve (AUROC) and area under the precision-recall curve (AUPRC) metrics compared to these traditional methods.

Another GNN-based method for cell-cell communication analysis in spatial transcriptomics, spaCI [3], employs a graph attention mechanism to model ligand-receptor interactions by integrating intracellular signaling and intercellular spatial information. In Fig. 2 of their study, the authors demonstrated that spaCI outperformed four popular traditional methods—iTALK [4], Connectome [5], CellChat [6], and CellPhoneDB [7]—in identifying ligand-receptor pairs, achieving the highest F1 score on simulated cohorts. Additionally, spaCI identified the highest number of overlapping ligand-receptor interactions compared to these methods on two real datasets: MERSCOPE colon tumor spatial data (https://info.vizgen.com/merscope-ffpe-solution) and NanoString CosMx data [8].

Besides, numerous other relevant benchmarks have been conducted. For instance, in the context of gene regulatory network (GRN) inference, CEFCON—a graph attention network (GAT)-based method—was evaluated against six traditional GRN construction methods, including SCINET [9], NetREX [10], CellOracle [11], GRNBoost2 [12], DeepSEM [13], and Random_NicheNet (which randomly selects edges from a prior gene interaction network). The performance of these methods was assessed on nine benchmark scRNA-seq datasets by metrics such as AUPRC and the early precision ratio (EPR). As shown in Fig. 3a of their article, CEFCON demonstrated significant advantages over traditional methods in constructing cell-lineage-specific GRNs. Similarly, scGCN [14] demonstrated enhanced cell type annotation accuracy compared to widely used Seurat v3 [15], while scGNN [16] showed superior imputation performance relative to scImpute [17] and scVI [18].

In addition to comparisons with traditional methods tailored for specific single-cell data applications, some GNN-based approaches are also evaluated against general traditional machine learning methods. For instance, in terms of dimensionality reduction, certain methods are compared with traditional techniques such as t-Distributed Stochastic Neighbor Embedding (t-SNE) [19] and principal component analysis (PCA). A GAT-based method, scGAE [20], demonstrates its ability to distinguish cell subpopulations by visualizing the generated low-dimensional embeddings, outperforming methods like t-SNE, SAUCIE [21], Ivis [22], and PHATE [23]. Furthermore, scGAE quantitatively compares its performance in cell clustering and trajectory inference against traditional methods such as PCA, DCA [24], and scVI, highlighting its advantages in capturing cellular heterogeneity.

In summary, GNN-based approaches have demonstrated superior performance compared to traditional methods, including those specifically designed for single-cell data analysis and general traditional machine learning techniques.

**Text S2. Graph convolutional networks (GCNs)**

Convolutional Neural Networks (CNNs) have been successfully applied in various fields, including text recognition [25], image classification [26, 27] and deconvolution [27], and emotion [27] and action [28] classification in video clips. However, traditional convolution operations, which have already achieved great success in the aforementioned applications, are defined on data with a fixed and regular structure, such as the pixels in an image, which are organized in a uniform grid. In contrast, graph data is made up of nodes and edges with flexible and non-uniform connections. Therefore, it is necessary to redefine convolution in the context of graphs. Bruna *et al*. [29] were the first to propose generalizing the convolution operator to data represented by graphs. One important approach in GCN is the spectral-based GCN, which leverages the spectral properties of graphs. Here, we briefly review a representative spectral-based GCN.

For undirected graph, Kipf and Welling proposed a multi-layer spectral-based GCN [30] for semi-supervised node classification in graph-structured data (Fig. 2A). The normalized graph Laplacian matrix is defined as $\mathbf{L}=\mathbf{I}_{N_{v}}-\mathbf{D}^{\mathbf{-}\frac{\mathbf{1}}{\mathbf{2}}}\mathbf{A}\mathbf{D}^{\mathbf{-}\frac{\mathbf{1}}{\mathbf{2}}}$. Let $\mathbf{U}$ denote the matrix of eigenvectors of $\mathbf{L}$ then $\mathbf{L}=\boldsymbol{U\Lambda}\mathbf{U}^{\mathbf{T}}$, where $\boldsymbol{\Lambda}$ is a diagonal matrix composed of eigenvalues of $\mathbf{L}$. The spectral convolution on a graph can be defined as Eq. (1) by the node feature vector $x\in\mathbb{R}^{N_{v}}$ and a learnable filter matrix $\mathbf{g}_{\boldsymbol{\omega}}=diag\left( \omega\right)$, where $\omega\in\mathbb{R}^{N_{v}}$ is the parameter.

$$\begin{aligned} \mathbf{g}_{\boldsymbol{\omega}}\star x=\mathbf{U}\mathbf{g}_{\boldsymbol{\omega}}\mathbf{U}^{\mathbf{T}}x.\#\left( 1 \right) \end{aligned}$$

To enhance computational efficiency, $\mathbf{g}_{\boldsymbol{\omega}}$ can be approximated by a truncated Chebyshev polynomial expansion up to the $M^{th}$ order according to [31] as follows:

$$\begin{aligned} \mathbf{g}_{\boldsymbol{\omega}}\left( \boldsymbol{\Lambda} \right)\approx\sum_{m=0}^{M} \omega_{m}\mathbf{T}_{\boldsymbol{m}}\left( \tilde{\boldsymbol{\Lambda}} \right),\#\left( 2 \right) \end{aligned}$$

where $\mathbf{T}_{m}$ is the Chebyshev polynomial of order $m$ and $\tilde{\boldsymbol{\Lambda}}=\frac{2}{\lambda_{max}}\boldsymbol{\Lambda}-\mathbf{I}_{N_{v}}$, with $\lambda_{max}$ representing the largest eigenvalue of matrix **L**. Defining $\tilde{\mathbf{L}}=\frac{2}{\lambda_{max}}\mathbf{L}-\mathbf{I}_{N_{v}}$, Eq. (1) can be transformed into

$$\begin{aligned} \mathbf{g}_{\boldsymbol{\omega}}\star x\approx\sum_{m=0}^{M} \omega_{m}\mathbf{T}_{\boldsymbol{m}}\left( \tilde{\mathbf{L}} \right)x.\#\left( 3 \right) \end{aligned}$$

GCN limits $M=1$ and $\lambda_{max}\approx2$, simplifying Eq. (3) to

$$\begin{aligned} \mathbf{g}_{\boldsymbol{\omega}}\star x\approx\omega_{0}x+\omega_{1}\left( \mathbf{L}\boldsymbol{-}\mathbf{I}_{\boldsymbol{N}_{\boldsymbol{v}}} \right)x=\omega_{0}x-\omega_{1}\mathbf{D}^{\mathbf{-}\frac{\mathbf{1}}{\mathbf{2}}}\mathbf{A}\mathbf{D}^{\mathbf{-}\frac{\mathbf{1}}{\mathbf{2}}}x\#\left( 4 \right) \end{aligned}$$

GCN further simplifies Eq. (4) by constraining the parameters $\omega=\omega_{0}=-\omega_{1}$ to reduce model complexity and enhance numerical stability. To mitigate the problem of exploding or vanishing gradients, GCN introduces $\mathbf{I}_{N_{v}}+\mathbf{D}^{-\frac{\boldsymbol{1}}{\boldsymbol{2}}}\mathbf{A}\mathbf{D}^{-\frac{\boldsymbol{1}}{\boldsymbol{2}}}\to{{\tilde{\mathbf{D}}}^{-\frac{1}{2}}\tilde{\mathbf{A}}\tilde{\mathbf{D}}}^{-\frac{1}{2}}$, where $\tilde{\mathbf{A}}=\mathbf{A}+\mathbf{I}_{N_{v}}$ and ${\tilde{\mathbf{D}}}_{\mathrm{ii}}=\sum_{j} {\tilde{\mathbf{A}}}_{\mathrm{ij}}$. Ultimately, the propagation rule of GCN can be written as follows:

$$\begin{aligned} \mathbf{H}^{\left( k+1 \right)}=\sigma\left( \hat{\mathbf{A}}\mathbf{H}^{\left( k \right)}\mathbf{W}^{\left( k \right)} \right),\#\left( 5 \right) \end{aligned}$$

where $\hat{\mathbf{A}}={{\tilde{\mathbf{D}}}^{-\frac{1}{2}}\tilde{\mathbf{A}}\tilde{\mathbf{D}}}^{-\frac{1}{2}}$, $\mathbf{W}^{\left( k \right)}$ is the parameter matrix associated with layer $k$, and $\mathbf{H}^{\left( k \right)}$ is the convolved matrix of the $k$-th layer.

Although spectral-based GCNs provide a solid theoretical foundation and effectively leverage the graph’s global structure through the Laplacian’s spectral properties, they also have some limitations and practical challenges. One of the primary challenges is the reliance on the eigen-decomposition of the Laplacian matrix, which has a computational complexity of $O(N_{v}^{3})$, making it impractical for large-scale graphs. Additionally, spectral-based GCNs are not inherently transferable between different graphs, as the eigenvectors of the graph’s Laplacian matrix are specific to each graph structure, restricting their generalization capabilities when applied to graphs with varying topologies.

In summary, GCNs are powerful yet computationally demanding GNN variants for defining convolution operations on graphs, providing the foundation for many subsequent advancements in graph neural network research.

**Text S3. GraphSAGE**

The computation process of GCN involves the Laplacian matrix $\mathbf{L}$, which relies on the adjacency matrix **A** specific to the entire graph and characterizes the graph structure, indicating that the test nodes must be present in the graph during training. Thus, GCN can be regarded as a transductive learning method on fixed graphs. However, if the test set contains graph structures not seen during training, such as new nodes, the Laplacian matrix $\mathbf{L}$ will change accordingly. As a result, the previously trained GCN may face difficulties in generalizing to these new structures, despite the importance of such generalization in many applications. To address the limitation of GCN’s inability to generalize to unseen nodes, Hamilton *et al*. proposed an inductive learning framework called GraphSAGE (Graph Sample and Aggregated) [32] for generating node embeddings in large graphs. Unlike GCN, which relies on the entire graph structure and requires all nodes (including test nodes) to be present during training, GraphSAGE can handle unseen nodes. GraphSAGE achieves this by taking node features as input (Fig. 2B) and using differentiable aggregator functions (such as mean, long short-term memory, or pooling aggregators) to combine information from neighboring nodes (Fig. 2C), which allows it to generate embeddings for new nodes not seen during training.

Another key concept of GraphSAGE is to perform uniform sampling of a fixed size (user-specified and variable per iteration) of each node’s neighbors, which serves as the neighborhood set for the forward propagation to limit the memory and runtime required for each iteration. Let $K$ be the number of network layers, which also determines how far each node can aggregate information from its neighbors. Specifically, each additional layer allows a node to collect information from its neighbors’ neighbors, extending the range of information aggregation. Thus, a $K$-layer network enables each node to gather information from nodes that are up to $K$ hops away. Let $\mathbf{W}^{\left( k \right)},\forall k\in\{1,\ldots,K\}$ denote a set of weight matrices, $AGG_{k},\forall k\in\{1,\ldots,K\}$ represent the $k$-th aggregator functions, $\sigma(\cdot)$ represent the nonlinear activation function, and $\mathcal{N}\left( v \right)$ denote the sampled neighborhood set of node $v$. GraphSAGE generates node embeddings in the following manner:

$$\begin{aligned} \boldsymbol{h}_{\mathcal{N}\left( v \right)}^{\left( k+1 \right)}=AGG_{k+1}\left( \{\boldsymbol{h}_{u}^{\left( k \right)},\forall u\in\mathcal{N}\left( v \right)\} \right)\#\left( 6 \right) \end{aligned}$$

$$\begin{aligned} \boldsymbol{h}_{u}^{\left( k+1 \right)}\boldsymbol{=}\sigma\left( \mathbf{W}^{\left( k+1 \right)}\boldsymbol{\cdot}\left( \boldsymbol{h}_{u}^{\left( k \right)}\parallel\boldsymbol{h}_{\mathcal{N}\left( v \right)}^{\left( k+1 \right)} \right) \right)\boldsymbol{\#}\left( 7 \right) \end{aligned}$$

where $\parallel$denotes concatenation. In these equations, $\boldsymbol{h}_{\mathcal{N}\left( v \right)}^{\left( k+1 \right)}$ represents the aggregated information from the neighbors of node $v$ at layer $k+1$, while $\boldsymbol{h}_{u}^{\left( k+1 \right)}$ represents the updated embedding for node $u$ after incorporating its own features and the aggregated features from its neighbors. This two-step process—first aggregating neighbor information and then updating node representations—ensures that each node’s embedding captures both its own information and the context provided by its local neighborhood.

In summary, GraphSAGE efficiently handles large-scale graphs by using neighborhood sampling and inductive learning, allowing it to generalize to unseen nodes without needing the entire graph structure. Additionally, GraphSAGE can update $\mathbf{W}^{\left( k \right)}$ and the parameters of $AGG_{k}$ through stochastic gradient descent in unsupervised or task-specific supervised settings. It can also operate in a minibatch setting, enabling it to scale to very large graphs. These features make GraphSAGE versatile and effective for embedding generation in large-scale graph scenarios.

**Text S4. Graph attention networks (GATs)**

GraphSAGE’s operation of sampling fixed-size neighborhoods may prevent access to the complete neighborhood. Additionally, when using the long short-term memory (LSTM) aggregator, GraphSAGE imposes a random order on the naturally unordered neighboring nodes in that the LSTM aggregator is not permutation invariant. To address these limitations, Veličković *et al*. introduced a GNN framework based on the self-attention mechanism for node classification, named graph attention networks (GATs) [33].

Unlike GraphSAGE, GAT incorporates the entire neighborhood set without fixed-size sampling, ensuring access to complete neighborhood information. Moreover, GAT does not assume any specific order of neighboring nodes, effectively maintaining permutation invariance. By learning to assign different attention weights to each neighbor, GAT can flexibly capture the relative importance of different neighbors, addressing the limitations of GraphSAGE in a more principled manner (Fig. 2D).

GAT takes the features of all nodes as input and generates the hidden states of nodes by learning to weigh the importance of each neighbor differently. Let $F$ represent the number of features per node, $a\in\mathbb{R}^{2F}$ denote a learnable weight vector, and $ATT:\mathbb{R}^{F}\times\mathbb{R}^{F}\mathbb{\to R}$ represent an attentional mechanism parametrized by $a$. To compute the contribution of the features of node $u$ to node $v$, also known as the attention coefficients $\alpha_{vu}^{\left( t+1 \right)}$, can be obtained by

$$\begin{aligned} \alpha_{vu}^{\left( t+1 \right)}=\frac{exp\left( LeakyReLU\left( a^{T}\left[ \mathbf{W}^{\left( t+1 \right)}h_{v}^{\left( t \right)}\parallel\mathbf{W}^{\left( t+1 \right)}h_{u}^{\left( t \right)} \right] \right) \right)}{\sum_{z\in\mathcal{N}\left( \mathcal{v} \right)} exp\left( LeakyReLU\left( a^{T}\left[ \mathbf{W}^{\left( t+1 \right)}h_{v}^{\left( t \right)}\parallel\mathbf{W}^{\left( t+1 \right)}h_{z}^{\left( t \right)} \right] \right) \right)},\#\left( 8 \right) \end{aligned}$$

where $LeakyReLU(\cdot)$ is a type of activation function that introduces a small slope for negative inputs, defined as $LeakyReLU(x)=max\left( x,\alpha x \right)$ and $\alpha$ is a small constant. Here, $W^{\left( t+1 \right)}$ is the weight matrix for layer $t+1$ and $h_{v}^{\left( t \right)}$ is the feature vector of node $v$ at layer $t$.

Then the hidden features of node $v$ is computed as

$$\begin{aligned} \boldsymbol{h}_{v}^{\left( t+1 \right)}=\rho\left( \sum_{z\in\mathcal{N}\left( \mathcal{v} \right)\cup\{\mathcal{v}\}} \alpha_{vu}^{\left( t+1 \right)}\mathbf{W}^{\left( t+1 \right)}h_{z}^{\left( t \right)} \right)\#\left( 9 \right) \end{aligned}$$

where $\rho(\cdot)$ represents a non-linear activation function. GAT is further enhanced by applying multi-head attention mechanism instead of self-attention mechanism. GAT is computationally efficient due to its parallelized computations, and is applicable to both transductive and inductive problems.

However, we note that GAT also has limitations, particularly in terms of scalability to very large graphs, as calculating attention coefficients for numerous edges can be computationally expensive. Moreover, with the widespread adoption of multi-head attention mechanism, the increased parameter count of GAT may also lead to overfitting when data is scarce. Despite these challenges, GAT remains a powerful tool for relational tasks, with ongoing research focusing on enhancing scalability and reducing computational costs to broaden its application scope.

**Text S5. Graph transformer networks (GTNs)**

GraphSAGE and GAT are both originally designed for homogeneous graphs, meaning they are only suitable for scenarios where all nodes and edges in the graph are of the same type. However, in many applications within single-cell omics, such as different types of cells, genes, and molecular interactions, graphs often exhibit heterogeneous properties, consisting of multiple types of nodes and edges that capture complex relationships. To address this limitation, Yun *et al*. proposed Graph Transformer Networks (GTNs) [34], which learn node embeddings for heterogeneous graphs in an end-to-end manner. GTNs identify useful multi-hop connections between nodes, known as meta-paths, to form new graphs and generate node representations via convolution operations on the new meta-path-based graphs.

The key component of GTNs is the differentiable graph transformation layer, which dynamically generates new graph structures during training. Let $\mathbf{X}\in R^{N\times F}$ be the input feature matrix, where $N$ is the number of nodes and $F$ is the number of features per node. GTNs learn to combine multiple adjacency matrices $\mathbf{A}_{\mathbf{1}},\mathbf{A}_{\mathbf{2}},\ldots,\mathbf{A}_{\boldsymbol{M}}$, where each $\mathbf{A}_{\boldsymbol{i}}\in R^{N\times N}$, into a new meta-path-based adjacency matrix $\mathbf{A}^{'}$:

$$\begin{aligned} \mathbf{A}^{\mathbf{'}}=\prod_{i=1}^{M} \mathbf{A}_{\boldsymbol{i}},\#\left( 10 \right) \end{aligned}$$

where $M$ is the number of layers. This new matrix $\mathbf{A}^{\mathbf{'}}$ is then used to generate new node embeddings. The use of multiple adjacency matrices allows GTNs to identify important paths, thus capturing complex relationships in heterogeneous data.

The convolution operation applied to the transformed graph can be represented as:

$$\begin{aligned} H^{(k+1)}=\sigma\left( \mathbf{A}^{\mathbf{'}}H^{\left( k \right)}\mathbf{W}^{\left( \boldsymbol{k} \right)} \right),\#\left( 11 \right) \end{aligned}$$

where $H^{(k)}$ represents the node embeddings at layer $k,$ $W^{(k)}$ is the learnable weight matrix, and $\sigma(\cdot)$ represents the nonlinear activation function. This operation enables GTNs to refine node embeddings based on both direct and indirect relationships derived from meta-paths.

GTNs are effective in tasks like cell-type identification and lineage reconstruction in single-cell omics, as they model complex heterogeneous interactions crucial for understanding cellular dynamics. However, they face challenges related to the interpretability of meta-path and computational complexity, especially when generating multiple candidate paths for large datasets. Despite these challenges, GTNs show promise in uncovering latent cellular patterns that traditional methods might miss.

**Text S6. Graph autoencoders (GAEs)**

GCNs, GraphSAGE, GATs, and GTNs primarily focus on the node classification task for graph-structured data, which limits their applicability to other important graph tasks. One of the key limitations is their inability to effectively learn latent representations for predicting relationships between nodes, such as link prediction, which is crucial for understanding connectivity patterns in networks. To address this limitation, Kipf and Welling extended the application of autoencoders and variational autoencoders to graph-structured data, named graph auto-encoder (GAE) and variational graph autoencoder (VGAE) [35], respectively. Here, we refer to the models proposed by these authors as GAE and VGAE to distinguish them from more general terms. Specifically, GAE employs a two-layer GCN as an encoder to yield latent embeddings for all nodes, denoted by $\mathbf{Z}\in\mathbb{R}^{N_{v}\times F}$, where $F$ represents the dimensionality of the node embeddings. The GCN is defined as

$$\begin{aligned} \mathrm{GCN}\left( \mathbf{X},\mathbf{A} \right)=\tilde{\mathbf{A}}ReLU\left( \tilde{\mathbf{A}}\mathbf{X}\mathbf{W}_{0} \right)\mathbf{W}_{1}\#\left( 12 \right) \end{aligned}$$

where $\mathbf{X}\in\mathbb{R}^{N_{v}\times d}$ is the feature matrix of the nodes, $d$ denotes the dimensionality of the node features. The modified adjacency matrix $\tilde{\mathbf{A}}$ is defined as $\tilde{\mathbf{A}}={\mathbf{D}^{-\frac{1}{2}}\mathbf{AD}}^{-\frac{1}{2}}$, and the ReLU function is given by $ReLU\left( \cdot\right)=max\left( 0,\cdot\right)$. Both $\mathbf{W}_{0}$ and $\mathbf{W}_{1}$ represent the weight matrices.

The representation $\mathbf{Z}$ is then obtained by $\mathbf{Z}=GCN\left( \mathbf{X},\mathbf{A} \right)$. GAE employs the inner product as the decoder to reconstruct the adjacency matrix $\hat{\mathbf{A}}=\sigma\left( \mathbf{ZZ}^{T} \right)$, where $\sigma\left( \cdot\right)$ is the logistic sigmoid function. To ensure the reconstructed adjacency matrix $\hat{\mathbf{A}}$ closely resembles the original $\mathbf{A}$, GAE applies binary cross-entropy as the loss function during training. Once $\mathbf{W}_{0}$ and $\mathbf{W}_{1}$ are established, the embeddings $\mathbf{Z}$ are fixed for a given $\mathbf{X}$ and $\mathbf{A}$.

However, GAEs also face challenges, particularly in terms of scalability when dealing with very large graphs, as the inner product decoder and binary cross-entropy loss can become computationally expensive. Despite these limitations, GAEs are widely used due to their simplicity and effectiveness in various graph-related tasks. Future research may focus on improving the scalability of GAEs, enhancing their ability to capture more complex graph structures, integrating more advanced decoding mechanisms to better reconstruct graph information, and exploring more efficient and advanced loss functions to further enhance their performance.

**Text S7. Variational graph autoencoders (VGAEs)**

Although GAEs are effective at learning node representations, they rely on deterministic encoders, which limits their ability to model uncertainty in the latent space. This can be a significant drawback when dealing with complex graph structures or incomplete data, where capturing uncertainty is crucial. To address this limitation, the embedding $\mathbf{Z}$ of VGAE is not derived from a deterministic function but is sampled from a learned Gaussian distribution (Fig. 2E). VGAE utilizes two GCNs, as defined by Eq. (12), as encoders to learn the mean $\boldsymbol{\mu}$ and variance $\boldsymbol{\sigma}$ of the multivariate Gaussian distribution as follows.

$$\begin{aligned} \boldsymbol{\mu}=\mathrm{GC}N_{\boldsymbol{\mu}}\left( \mathbf{X},\mathbf{A} \right)\boldsymbol{\#}\left( 13 \right) \end{aligned}$$

$$\begin{aligned} log\boldsymbol{\sigma}=\mathrm{GC}N_{\boldsymbol{\sigma}}\left( \mathbf{X,A} \right).\#\left( 14 \right) \end{aligned}$$

Here, $\mathrm{GC}N_{\boldsymbol{\mu}}$ and $\mathrm{GC}N_{\boldsymbol{\sigma}}$ share the parameter $\mathbf{W}_{0}$ for initial feature extraction but use separate $\mathbf{W}_{1}$ parameters to independently learn the characteristics of the mean and variance, respectively. Then, the stochastic latent variables $\boldsymbol{z}_{i}$ and the corresponding node embedding matrix $\mathbf{Z}\in\mathbb{R}^{N_{v}\times F}$ composed of $\boldsymbol{z}_{i}$ can be sampled from a multivariate Gaussian distribution defined by $\boldsymbol{\mu}$ and $\boldsymbol{\sigma}$. This sampling process allows the model to effectively capture uncertainty in the latent space. The distribution for each latent variable $z_{i}$ is defined as:

$$\begin{aligned} q\left( \boldsymbol{z}_{i} \mid\mathbf{X},\mathbf{A} \right)\mathcal{=N}\left( \boldsymbol{z}_{i} \mid\boldsymbol{\mu}_{i},diag\left( \boldsymbol{\sigma}_{i}^{2} \right) \right).\#\left( 15 \right) \end{aligned}$$

To generate the node embedding matrix $\mathbf{Z}$, which consists of all node embeddings, VGAE assumes independence across nodes, leading to the joint probability distribution:

$$\begin{aligned} q\left( \mathbf{Z} \mid\mathbf{X,A} \right)=\prod_{i=1}^{N_{v}} q\left( \boldsymbol{z}_{i} \mid\mathbf{X,A} \right).\#\left( 16 \right) \end{aligned}$$

The decoder of VGAE reconstructs the adjacency matrix by taking the inner product of latent variables as follows:

$$\begin{aligned} p\left( \mathbf{A} \mid\mathbf{Z} \right)=\prod_{i=1}^{N_{v}} \prod_{j=1}^{N_{v}} p\left( \mathbf{A}_{ij} \mid\boldsymbol{z}_{i},\boldsymbol{z}_{j} \right),\#\left( 17 \right) \end{aligned}$$

where $p\left( \mathbf{A}_{ij}=1 \mid\boldsymbol{z}_{i},\boldsymbol{z}_{j} \right)=\sigma\left( \boldsymbol{z}_{i}^{T}\boldsymbol{z}_{j} \right)$ and $\sigma\left( \cdot\right)$ is the same activation function as used in GAE. VGAE utilizes the following loss function:

$$\begin{aligned} \mathcal{L=-}E_{q\left( \mathbf{Z} \mid\mathbf{X},\mathbf{A} \right)}\left[ \log p\left( \mathbf{A} \mid\mathbf{Z} \right) \right]+KL\left[ q\left( \mathbf{Z} \mid\mathbf{X},\mathbf{A} \right)\parallel p\left( \mathbf{Z} \right) \right],\#\left( 18 \right) \end{aligned}$$

where $KL\left[ q\left( \cdot\right)\parallel p\left( \cdot\right) \right]$ represents the Kullback-Leibler divergence between the probability distributions $q\left( \cdot\right)$ and $p\left( \cdot\right)$. The reparameterization trick is applied to address the issue of unavailable gradients caused by sampling operations.

In summary, VGAE’s key advantage over GAE lies in its ability to model uncertainty, making it suitable for incomplete or noisy graphs. However, it introduces greater computational complexity and challenges in ensuring meaningful latent representations. Future work could focus on scalability and improving the interpretability of latent variables.

**Text S8. Message passing neural networks (MPNNs)**

MPNNs are a framework designed for learning on graph-structured data [36, 37]. The framework operates by iteratively updating node representations through two main phases: the message passing phase and the readout phase. In the message passing phase, each node in the graph updates its hidden state by aggregating messages from its neighbors. These messages are computed by a message function, which takes into account both the node features and the edge features between neighboring nodes. Mathematically, the message passed to node 𝑣 at time step 𝑡+1 is calculated as the sum of messages from all its neighbors:

$$\begin{aligned} \mathbf{m}_{v}^{(t+1)}=\sum_{w\in N(v)} M_{vw}(\mathbf{h}_{v}^{(t)}, \mathbf{h}_{w}^{(t)},\mathbf{e}_{vw}),\#\left( 19 \right) \end{aligned}$$

where 𝑁(𝑣) represents the set of neighbors of node 𝑣, $\mathbf{h}_{v}^{(t)}$ and $\mathbf{h}_{w}^{(t)}$ are the hidden states of nodes 𝑣 and 𝑤, respectively, and $\mathbf{e}_{vw}$ is the feature associated with the edge between these two nodes. The message function $M_{vw}$ can be designed to capture the specific interactions between nodes and edges, making it flexible for various tasks.

Once the messages have been computed, the hidden states of the nodes are updated using a vertex update function $U_{t}$. This function combines the current hidden state of a node $\mathbf{h}_{v}^{(t)}$ with the aggregated message $\mathbf{m}_{v}^{(t+1)}$to produce the updated hidden state $\mathbf{h}_{v}^{(t+1)}$. The update process is expressed as:

$$\begin{aligned} \mathbf{h}_{v}^{(t+1)}=U_{t}\left( \mathbf{h}_{v}^{\left( t \right)},\mathbf{m}_{v}^{\left( t+1 \right)} \right).\#\left( 20 \right) \end{aligned}$$

After the message passing and vertex update phases, the readout phase computes a global representation of the entire graph. This is done by aggregating the final hidden states of all nodes in the graph using a readout function 𝑅. The global representation of the graph $\hat{\mathbf{y}}$ can be calculated as:

$$\begin{aligned} \hat{\mathbf{y}}=R\left( \left\{ \mathbf{h}_{v}^{(T)}|v\in G \right\} \right),\#\left( 21 \right) \end{aligned}$$

where $\mathbf{h}_{v}^{(T)}$ is the final hidden state of node 𝑣 after 𝑇 time steps, and 𝐺 represents the entire graph. The readout function is designed to aggregate node information into a fixed-size representation of the entire graph, which can be used for tasks such as graph classification or regression.

MPNNs are highly flexible, as the message functions $M_{vw}$, vertex update functions $U_{t}$, and readout functions 𝑅 can be chosen according to the specific requirements of the task. For instance, some MPNN implementations use self-attention mechanisms during the message aggregation step, allowing the model to focus on more important neighbors. This flexibility enables MPNNs to handle various graph types, including undirected and directed graphs, and adapt to different types of features.

**Text S9. Differences between GATs and GTNs**

Although both GAT [33] and GTN [34] utilize attention mechanisms, specifically quadratic attention mechanisms within global attention frameworks, they differ significantly. In this revision, we have summarized the differences between GAT and GTN in terms of the types of graphs they can model and the roles of their attention mechanisms.

Firstly, regarding the graph structures they can model, GAT can only handle homogeneous graphs, whereas GTN can operate on heterogeneous graphs, which are capable of modeling complex graphs consisting of various types of nodes and edges.

Secondly, the objects weighted by the attention coefficients differ between GAT and GTN.

For GAT, the input consists of the features of all the nodes in the graph. In each graph attention layer, GAT applies a shared weight matrix $\mathbf{W}\in\mathbb{R}^{F^{'}\times F}$ to linearly transform the features of each node, where $F$ represents the length of the input feature vector of each node, and $F^{'}$ denotes the length of the output feature vector of each node at this layer. The graph attention layer then computes self-attention between nodes, that is, the attention coefficients between the linearly transformed node features, which represent the importance of one node’s feature relative to another. Notably, GAT employs masked attention, calculating attention coefficients only between neighboring nodes (typically first-order neighbors) rather than all node pairs, thereby preserving the structural information. The attention coefficients, after softmax normalization, are used as weights to compute the weighted sum of the linearly transformed node features. A non-linear activation function is then applied to this weighted sum to obtain the final output feature for each node. For more details, we refer the readers to “Supplementary Text S4: Graph attention networks (GATs)” and the original paper for GAT. In essence, GAT utilizes attention coefficients to weight node features and, through this self-attention-based approach, focuses on neighboring nodes to compute node embeddings.

For GTN, it considers the input graph $\mathcal{G}$ as a heterogeneous graph, where the number of edge types is greater than 1. Let $\mathcal{T}^{e}$ be the set of edge types, and $K=\left| \mathcal{T}^{e} \right|$. The heterogeneous graph $\mathcal{G}$ can be represented by a set of adjacency matrices ${{\{\mathbf{A}}_{k}\}}_{k=1}^{K}$, which can also be written as $\mathbb{A\in}\mathbb{R}^{N\times N\times K}$, where $\mathbf{A}_{k}\in\mathbb{R}^{N\times N}$, $N$ is the number of nodes in the graph, and $\mathbf{A}_{k}\left[ i,j \right]\neq0$ if an edge of type $k$ connects node $v_{j}$​ to node $v_{i}$. A meta-path $p$ is a multi-hop connection $\begin{aligned} v_{1}\overset{t_{1}}{\to}v_{2}\overset{t_{2}}{\to}\ldots\overset{t_{l}}{\to}v_{l+1} \end{aligned}$, composed of heterogeneous edges in the heterogeneous graph $\mathcal{G}$, where $t_{l}\in\mathcal{T}^{e}$ represents the $l$-th edge type of the meta-path. Given a sequence of edge types $\left( t_{1},t_{2},\ldots,t_{l} \right)$, the adjacency matrix $\mathbf{A}_{p}$ of the meta-path $p$ can be obtained by multiplying the corresponding adjacency matrices: $\mathbf{A}_{p}=\mathbf{A}_{t_{l}}\ldots\mathbf{A}_{t_{2}}\mathbf{A}_{t_{1}}$. Accordingly, a new graph structure can be defined with meta-path $p$ as the edge. GTN essentially learns node embeddings by performing convolution on the meta-path graphs learned from the Graph Transformer (GT) layers on the heterogeneous graph. The GT layer consists of two parts. First, it selects two graph structures $Q_{1}$ and $Q_{2}$ from $\mathbb{A}$, with each adjacency matrix $Q=F\left( \mathbb{A;}W_{\phi} \right)=\phi\left( \mathbb{A};softmax\left( W_{\phi} \right) \right)$, where $\phi$ is the convolution layer and $W_{\phi}\in\mathbb{R}^{1\times1\times K}$ is the weight parameter of $\phi$. Each $Q_{i}$ can be represented as $\sum_{t_{l}\in\mathcal{T}^{e}} \alpha_{t_{l}}^{\left( l \right)}\mathbf{A}_{t_{l}}$, where $\alpha_{t_{l}}^{\left( l \right)}$ is the weight and the attention score of edge type $t_{l}$ at the $l$-th GT layer. Then, the adjacency matrix for any meta-path $P$ of any length $l$ can be expressed as $\mathbf{A}_{P}=\left( \sum_{t_{1}\in\mathcal{T}^{e}} \alpha_{t_{1}}^{(1)}\mathbf{A}_{t_{1}} \right)\left( \sum_{t_{2}\in\mathcal{T}^{e}} \alpha_{t_{2}}^{(2)}\mathbf{A}_{t_{2}} \right)\ldots\left( \sum_{t_{l}\in\mathcal{T}^{e}} \alpha_{t_{l}}^{(l)}\mathbf{A}_{t_{l}} \right)$. The weight of the meta-path $t_{l},t_{l-1},\ldots,t_{0}$ is $\prod_{i=0}^{l} \alpha_{t_{i}}^{(i)}$, which is also the attention score of the meta-path and indicates its importance in the overall prediction task. In conclusion, GTN utilizes the attention score to weight the adjacency matrices.

**Text S10.** **An overall introduction to the single-cell-processing pipeline for each application and the roles of GNNs in these pipelines**

**Dimensionality reduction.** Dimensionality reduction plays a crucial role in the analysis of single-cell transcriptomics data, as scRNA-seq data is often characterized by high dimensionality and noise, complicating subsequent analyses. By reducing the dimensionality, the inherent complexity of single-cell omics data is simplified, facilitating the identification of meaningful biological patterns. GNN-based methods have proven to be particularly effective for this purpose, enabling the extraction of low-dimensional representations of cells that capture their intricate relationships. These representations can then be leveraged for a variety of tasks, including cell clustering, gene imputation, and cell type classification. For SRT data, dimensionality reduction also plays a key role. By projecting the data into a lower-dimensional space, researchers can capture spatial heterogeneity more effectively, which is critical for downstream analyses, such as identifying spatial domains, inferring spatial patterns, and correcting batch effects. This technique provides a powerful tool for analyzing and interpreting spatial transcriptomic data. Across the methods we reviewed, GNNs are commonly applied to cell-cell graphs, spot-spot graphs, or cell-gene-loci graphs for dimensionality reduction, aiming to learn low-dimensional embeddings.

**Imputation.** Imputation is a crucial task in the analysis of single-cell omics data. The main goal of imputation is to recover missing, unmeasured, or sparse values which often resulted from technical noise, low sequencing depth, or other experimental limitations in single-cell datasets. Imputation helps to improve the completeness and quality of data for downstream analyses, such as clustering, differential expression analysis, and trajectory inference. In the 107 methods we reviewed, GNNs are commonly applied to cell-cell graphs, gene-gene graphs, cell-locus graphs, or chromosomal segment-chromosomal segment graphs for imputing single-cell omics data.

**Cell type identification.** Cell type identification is a fundamental application in the analysis of scCAS data, enabling the exploration of complex, cell-type-specific gene regulatory mechanisms and enhancing our understanding of cellular functions and their alterations in health and disease states. In terms of methodologies, cell type identification can be divided into two main approaches: clustering and classification. Single-cell clustering approaches can be categorized into two primary types. The first type involves dimensionality reduction using traditional or deep learning methods to obtain low-dimensional embeddings, followed by the application of clustering algorithms such as K-means, Louvain, or Leiden on these embeddings. The second type focuses on calculating or learning the similarity between cells and clustering based on these similarity metrics. In single-cell analysis, cell classification is a crucial task, and there are various methods available for cell annotation. One such approach is label transfer, which involves mapping cell types from a reference dataset to a query dataset. This method is particularly useful when a well-annotated reference atlas is available, allowing for the prediction of cell types in new, unannotated datasets. Across the methods we reviewed, GNNs are frequently employed on cell-cell graphs, gene-gene graphs, gene-cell graphs, or prototype-prototype-cell graphs for cell type identification.

**Gene regulatory network (GRN) inference.** GRN inference can be regarded as a link prediction task between genes, aiming to elucidate the complex gene interactions that govern cellular functions. GRN inference also encompasses branches focusing on specific gene regulatory mechanisms, such as transcription factor regulatory network inference. For example, DeepTFni [38] employs a VGAE to infer transcription factor regulatory networks from scCAS data. GNNs are frequently utilized on gene-gene graphs to perform GRN inference.

**Knockout-responsive gene prediction.** Predicting knockout-responsive genes involves predicting the changes in the expression levels of other genes following the knockout of a specific gene. GNNs are implemented on gene-gene graphs for this purpose.

**Cell-cell communication inference.** Cell-cell communication inference usually involves identifying ligand-receptor interactions and weighting these interactions to reconstruct multicellular intra- and intercellular communication networks. GNNs are commonly applied to gene-gene graphs, cell-cell graphs, or protein-protein graphs to reconstruct these communication networks.

**Disease state prediction.** In single-cell omics research, disease state prediction aims to utilize single-cell data to forecast an individual’s health status or disease condition. This task is crucial for early diagnosis, personalized treatment, and understanding disease mechanisms. In the studies we reviewed, GNNs are typically applied to cell-cell graphs for disease state prediction.

**Perturbation prediction.** Single-cell perturbation prediction involves analyzing single-cell data to forecast or simulate changes in cellular features—such as gene expression, protein levels, and metabolism—under specific perturbations like gene activation, drug treatment, or environmental changes. This task is crucial for elucidating cellular responses to external or internal stimuli and provides valuable biological insights for gene function studies, disease therapies, and drug development. In the methods we reviewed, GNNs have been employed on gene-gene or cell-cell graphs to predict single-cell perturbation.

**Immune response prediction.** Immunity response prediction aims to predict or simulate the response of the immune system to specific stimuli. This can be achieved by constructing a cell-cell graph from single-cell data and applying GNNs, as demonstrated in SNOWFLAKE [39], to model the immune response.

**Single-cell DNA methylation level prediction.** We collected only one study that applies GNN to predict single-cell DNA methylation levels, namely scHiMe [40], which predicts base-pair-specific methylation levels in individual cells using scHi-C data and DNA nucleotide sequences. scHiMe employs the metacell strategy and utilizes a GTN-based model on the promoter-promoter spatial interaction network to predict DNA methylation levels.

**Spatial gene expression prediction.** The prediction pipeline for spatial gene expression involves constructing cell-cell or spot-spot *k*NN graphs based on Euclidean distance. Additionally, it can integrate data from other modalities, such as histology images, to predict jointly.

**Multi-omics integration.** Multi-omics data integration aims to jointly analyze different omics data from the same cell to obtain a more comprehensive view. The typical pipeline for multi-omics integration often involves projecting data from different omics into a shared lower-dimensional space. In the studies we reviewed, GNNs are applied to feature-feature graphs or cell-cell graphs.

**Granger causal inference and biological network inference.** These two types of tasks are highly specific and involve diverse scenarios, making it difficult to generalize. Among the 107 articles we reviewed, five studies fall into these categories. These studies constructed various types of graphs, such as cell-cell, gene-gene, and disease-gene graphs, or combinations thereof, to perform inference.

**Cell type deconvolution.** Cell type deconvolution aims to estimate the proportions of different cell types within each spot. This process is typically performed based on a spot-spot graph.

**Multiple SRT slices integration**. Integrating multiple SRT slices involves aligning adjacent tissue sections from various orientations to achieve a comprehensive spatial map of gene expression. This integration can be effectively performed by constructing a spot-spot graph, which transforms the data into a graph structure that can be processed by GNNs.

**Protein abundance prediction.** Protein abundance prediction can be achieved by constructing heterogeneous graphs such as cell-gene, gene-protein, gene-gene, and protein-protein graphs based on multi-omics data, or by integrating a protein-protein interaction (PPI) network as prior knowledge to build a protein-protein graph and applying GNNs.

**Cell-wise fluxome estimation.** Cell-wise fluxome estimation aims to quantify the flow of metabolites through metabolic pathways at the single-cell level. Fluxomics focuses on mapping the rate of biochemical reactions and how metabolites are produced and consumed within each cell. Estimating these fluxes offers valuable insights into cellular activities that are not fully captured by gene expression data alone, such as energy production efficiency and metabolic adaptation. Cell-wise fluxome estimation can be achieved by applying GNNs on a directed factor graph formulated from the metabolic network.

**Text S11. GNNs for single-cell transcriptomics**

**Imputation.** Self-supervised learning has been explored to enhance the imputation process. scGCL [41] incorporates a self-supervised graph representation learning method named AFGRL [42], which utilizes a GCN as the encoder combined with a contrastive learning strategy to impute dropouts. In addition, a zero-inflated negative binomial (ZINB) based autoencoder is used to reconstruct the gene expression matrix, improving the robustness of the final imputation.

**Dimensionality reduction.** To capture different aspects of cellular relationships, some methods use multiple graph types, allowing for a comprehensive view of the data. For example, CellVGAE [43] incorporates Pearson correlation graphs in addition to kNN graphs to capture multiple types of cell relationships. It employs an unsupervised variational graph attention autoencoder to learn low-dimensional embeddings, which are then used for cell classification via HDBSCAN [44] clustering algorithm, leveraging both gene expression similarity and structural information from the graph.

Hybrid approaches combine different neural network layers or data modalities to enhance model performance [45-48]. For example, scGNN 2.0 [45] optimizes the original scGNN by integrating a graph attention autoencoder and incorporating bulk RNA-seq data, thereby improving both clustering and imputation accuracy.

Building upon the framework introduced by scGNN, scGAE [91] enhances the embedding process by incorporating a GAT encoder, which allows the model to assign different weights to neighboring nodes, thus capturing more nuanced relationships. To further ensure that both structural and feature-level information is preserved, scGAE utilizes two decoders for reconstructing both the expression and adjacency matrices.

Graph attention mechanisms assign different weights to neighboring nodes, allowing GAT-based models to focus on the most relevant relationships in the data [94-96]. For example, SCDRHA [94] first uses a deep count autoencoder (DCA) [97] to denoise scRNA-seq data before applying a GAT encoder on the cell kNN graph to learn low-dimensional embeddings, effectively capturing cell relationships.

**Cell type identification.** Focusing on gene interactions, these approaches use graphs where nodes represent genes, providing a deeper understanding of cellular functions [49, 50]. For example, sigGCN [49] incorporates prior gene-gene interactions to construct a gene graph, combining GCN and a fully connected neural network for cell classification. Moreover, some approaches utilize heterogeneous graphs that represent different types of nodes or relationships to capture more complex biological structures [3, 51]. For instance, CAME [3] constructs a heterogeneous graph involving both genes and cells from two different species, facilitating cross-species cell type annotation.

The choice of learning framework—self-supervised, semi-supervised, or unsupervised—affects how cell type annotations are generated. Each of these methods leverages unique strategies to enhance data representation. As for self-supervised and unsupervised methods, these approaches can work without labeled data, making them highly suitable for scRNA-seq analysis where annotated data is often scarce [97-99]. For example, graph-sc [97] constructs a gene-cell graph from gene expression data and uses a GAE to learn embeddings, which are subsequently used for unsupervised clustering. In the aspect of learning strategy, some approaches focus specifically on optimizing the clustering and classification process through iterative graph updates, advanced decoders, or contrastive learning [100-102]. Methods like scGAC [100] incorporates a dynamic iterative process, initially denoising the similarity matrix and subsequently feeding it into a VGAE for representation learning and clustering, further improving the accuracy and quality of the resulting cell embeddings.

Besides, semi-supervised approaches are particularly useful when a well-annotated reference dataset is available, enabling more accurate label transfer [52-54]. For instance, scDeepSort [52] uses a pre-trained GraphSAGE-based model to classify cell types without relying on external references, employing a weighted cell-gene graph for effective classification. Besides, some methods integrate various network types, such as autoencoders or other deep learning layers, to improve representation learning [55-57]. GDEC [55], for instance, employs a GCN for feature extraction, which is then combined with deep embedding clustering to facilitate cell clustering across different species through transfer learning.

Moreover, contrastive learning and transfer learning strategies can significantly enhance the robustness of cell embeddings. For instance, scDGDC [58] leverages dual-GCN encoders combined with a contrastive learning module to simultaneously optimize both cell embeddings and clustering, ensuring high-quality representations.

**Gene regulatory network (GRN) inference.** GRNs represent the complex interactions among genes that regulate gene expression. Inferring GRNs from scRNA-seq data is essential for understanding cellular behavior, such as differentiation, as well as identifying key regulators that control specific cellular processes. Below, we discuss several GNN-based approaches developed for GRN inference, highlighting the different graph construction techniques and learning strategies employed to tackle this challenge.

Methods such as CEFCON [97] integrate prior information to address the inherent challenges of high sparsity and noise in scRNA-seq data. Taking scRNA-seq data as input, CEFCON employs NicheNet [98] to generate a gene interaction network, which is then presented as a directed graph with genes as nodes. Subsequently, CEFCON utilizes a GAT to derive representations of genes, where the GAT is trained through the deep graph infomax (DGI) [99] contrastive learning strategy. The acquired attention coefficients are then applied as weights to prioritize edges within the prior network, thus constructing the cell-lineage-specific GRN. This approach leverages prior knowledge to better capture biologically relevant interactions.

Another approach for GRN inference involves using GAEs to learn latent representations of genes and predict potential regulatory links. DeepRIG [100] constructs a weighted gene co-expression network from gene expression profiles and further yields a gene prior regulatory graph which is subsequently integrated into a GAE framework to infer GRNs.

GNNLink [59] takes advantage of external biological databases to construct an initial gene interaction graph. This initial graph serves as input for a GCN-based encoder, which learns gene features that can reconstruct the gene graph through link prediction. The inferred regulatory relationships are derived by computing the dot products of gene features, thus capturing latent interactions among genes. This method benefits from leveraging well-established biological data as a foundation for inferring new regulatory links, enhancing the reliability of the inferred GRN.

**Knockout-responsive gene prediction.** Predicting gene functions without knockout (KO) samples is a challenging task in single-cell transcriptomics data analysis, as KO experiments are costly and time-consuming. The goal of knockout-responsive gene prediction is to infer the effects of gene deletions computationally using wild-type data, enabling insights into gene functions without requiring experimental KO data.

To address the challenge of predicting gene functions without KO samples, Yang *et al.* [60] proposed GenKI, a VGAE-based model. GenKI takes scRNA-seq profiles of wild-type samples as input. It first constructs an incomplete single-cell gene regulatory network (scGRN) using principal component regression [61] and selectively filters its edges. Then it converts the processed scGRN into a binary gene-by-gene adjacency matrix, which serves as input for the VGAE model.

GenKI learns latent representations of genes using the VGAE model. To simulate the knockout of a target gene, it sets the weights of all target gene-related edges in the scGRN to zero, effectively removing the influence of that gene. The modified scGRN is then input to the pretrained VGAE encoder to generate new gene embeddings. The impact of the knockout is assessed by calculating the KL divergence between gene distributions before and after the knockout. This helps in understanding the potential functions of the knocked-out gene.

By incorporating virtual knockout simulations, GenKI enables researchers to infer gene functions and explore regulatory relationships efficiently, providing a valuable alternative to traditional knockout experiments.

**Disease state prediction.** Disease state prediction aims to classify individual cells into different health states using scRNA-seq data, helping to diagnose and understand diseases like autoimmune conditions and cancers. Ravindra *et al.* [62] introduced a GAT-based model (referred to as Ravindra_2020) to predict disease state from scRNA-seq data. This model first constructs a cell neighborhood graph using *k*NN and then applies a GAT to predict the probability that a cell belongs to a Multiple Sclerosis patient versus a healthy adult. The attention weights of GAT also provide low-dimensional representations for data visualization, making it easier to interpret the results. Sehanobish *et al.* [63] enhanced disease state prediction by introducing extra edge features to differentiate various disease states and severities, enhancing model interpretability with GNNExplainer [64] and attention mechanisms. Specifically, this model, referred to as Sehanobish_2021, employs BBKNN for cell graph construction, constructs a cell graph using BBKNN and employs a GAT to learn cell embeddings. Furthermore, Sehanobish_2021 generates new edge features through auxiliary tasks and integrates a Set Transformer [65] for node classification.

**Single-cell perturbation prediction.** Cellular transcriptional responses to genetic perturbations reveal fundamental principles of cell function [66]. Computational modeling of perturbation effects at single-cell resolution enables critical applications ranging from predicting cellular responses to environmental stress to forecasting patient-specific drug efficacy [67]. One of the central objectives of single-cell perturbation prediction is to develop computational frameworks to simulate complex gene-gene and gene-phenotype interactions, thereby enabling accurate predictions of cellular state alterations induced by novel perturbations (e.g., gene knockouts, overexpression, or combinatorial interventions). This emerging paradigm offers novel perspectives for understanding dynamic gene regulatory networks and demonstrates significant potential in drug development. GEARS [66] represents a computational breakthrough that addresses critical limitations of conventional experimental approaches constrained by the combinatorial explosion in multi-gene perturbation studies. For single-cell transcriptomic data, GEARS combines GNN technology with a knowledge graph of gene-gene relationships, applying a GNN to integrate information from neighboring genes within a gene co-expression graph. In this graph, nodes represent genes, and edges connect co-expressed genes. Given unperturbed single-cell gene expression and the perturbation set, GEARS predicts the transcriptional state of the cell following the perturbation of single genes or gene combinations of genes, where the prior experimental perturbation data is unavailable. Given single-cell transcriptomics data from multiple conditions, Cellograph [68] employs a GCN-based semi-supervised framework to learn latent cell embeddings based on how well each cell represents its true condition label. Besides, Cellograph learns a gene weight matrix that reveals the crucial genes directing the variations across conditions.

**Text S12. GNNs for spatially resolved transcriptomics**

**Spatial gene expression prediction.** Building on a similar concept of leveraging histological images, Cells2RNA [107] takes a more granular approach by focusing on individual cells within the histology images. Cells2RNA first employs a CNN to capture cell-level features in histological images and then utilizes GATs to learn cell embeddings on a spatial cell kNN graph constructed based on Euclidean distances. Unlike Hist2ST, which predicts gene expression at the spot level, Cells2RNA predicts gene expression at the cell level first and then aggregates these predictions to obtain spot-level gene expression.

**Dimensionality reduction.** Integrating additional data modalities for dimensionality reduction can yield more informative representations [109-111]. conST [109] constructs a cell or spot *k*NN graph based on the spatial information, integrating gene expression and accessible morphology as node features into a VGAE using the contrastive learning strategy and refines the low-dimensional embeddings through deep clustering via k-means. PAST [69] characterizes spatial domains based on a VGAE model, featuring a Bayesian neural network (BNN) to integrate prior knowledge from reference scRNA-seq datasets, along with a self-attention mechanism to identify spatial patterns. Additionally, PAST uses a ripple walk sampler to facilitate scalable training and prediction based on subgraphs.

**Spatial domain identification.** Extending on SpaGCN, STAGATE [115] further considers the adaptive learning of the similarity among neighboring spots and the spatial similarity at the boundaries of spatial domains. It uses an adaptive graph attention auto-encoder [123] to learn low-dimensional representations of spots or cells. These embeddings are clustered using mclust [124] or the Louvain clustering method to detect spatial domains. SpaceFlow [70] employs the deep graph infomax (DGI) [71] with a two-layer GCN as an encoder to yield spatially consistent low-dimensional cell embeddings for SRT data, or spot embeddings for SRT data. In this framework, each node represents a cell or a spot and edges depict the spatial neighborhood relationships between nodes. SpaceFlow then applied the Leiden algorithm to cluster these embeddings to identify spatial domains. Additionally, it runs the diffusion pseudotime [72] method on the embeddings to obtain a pseudo-Spatiotemporal Map (pSM), which aids in inferring cellular spatiotemporal pattern inference in tissues. CCST [73] constructs a hybrid adjacency matrix from the cell neighborhood graph, along with gene expression data, is input into a DGI network featuring a four-layer GCN as the encoder to derive node embeddings. Following principal component analysis (PCA), CCST utilizes the *k*-means++ algorithm for cell clustering.

SpaGCN, STAGATE, and CCST are all unsupervised methods that may exhibit suboptimal performance in delineating the boundaries of spatial domains. SpaceFlow, despite employing a self-supervised contrastive learning approach, fails to adequately consider the local context of spots instead of the global context of spots. Overcoming these limitations, GraphST [74] incorporates a GCN-based self-supervised contrastive learning framework to learn spot representations that account for local spatial context. GraphST also supports horizontal and vertical integration of tissue slices, as well as projection of scRNA-seq data onto SRT data, thereby improving spatial domain resolution and allowing for better cell-type proportion estimation within spots.

**Cell-cell communications inference.** NCEM [75] and spaCI [76] emphasize both intracellular and intercellular interactions. NCEM integrates GCN and GAT to infer intercellular communications, particularly ligand-receptor dynamics, using subcellular molecular profiling. It also models intracellular signaling, enhancing the understanding of functional cell responses within the local cellular environment. spaCI employs a graph attention mechanism to elucidate ligand-receptor interactions, thus integrating both intracellular signaling and intercellular spatial information to understand cell-cell communication. DeepLinc [41] constructs an undirected cell-cell neighborhood graph based on geometric closeness, where edges signify proximal relationships among cells. This graph, transformed into an adjacency matrix, along with spatial gene expression data, is fed into a VGAE-based model integrated with an adversarial network for regularization to learn latent cell embeddings that can be utilized for clustering and visualization. DeepLinc outputs a reconstructed cellular interaction network considering both distal and proximal interplays.

**Text S13. GNNs for single-cell multi-omics**

**Multi-omics integration.** Some methods extend these techniques by accounting for spatial relationships and multi-omics interactions [77, 78]. To bridge the gap in computational approaches for investigating diverse spatial data modalities in 3D, Zhang *et al.* developed STACI [77], an over-parameterized VGAE-based model to integrate multi-omics data including spatial transcriptomics paired with chromatin images and correct batch effects in the joint latent space. Taking each cell as a node, STACI determines the edges between cells based on the proximity of their spatial locations and learns representations incorporating gene expression and cell spatial coordinates for downstream analyses. It enables the prediction of gene expression from chromatin imaging and facilitates the integration of multiple SRT slices from diverse tissues.

Extending the integration capability to include proteomics data, GCN-SC [79] identifies cell pairs within and between the reference and query dataset based on the mutual nearest neighbors algorithm and implements a GCN-based model to integrate scRNA-seq data, scCAS data, and CITE-seq data and transfer labels. It then utilizes NMF for dimensionality reduction on the matrices generated from each omics.

**Granger causal inference.** Understanding causality at the molecular level is critical for deciphering the complex regulatory mechanisms governing cellular functions. Granger causal inference is a powerful tool for analyzing time-dependent relationships, enabling researchers to predict how changes in one variable can causally impact another over time. In single-cell multi-omics data, inferring Granger causal relationships can shed light on the dynamics between chromatin accessibility and gene expression.

For jointly profiled scCAS and scRNA-seq data, Singh et al. [42] proposed GrID-Net, a GNN-based framework, to infer Granger causal relationships between peaks and gene expression in a single cell. GrID-Net treats each cell as a node and links cells that exhibit resembling multi-omics profiles. The orientation of edges is established through pseudotime analysis, thereby constructing directed acyclic graphs (DAGs). By incorporating pseudotime, GrID-Net models the dynamic nature of chromatin accessibility and gene expression changes. To infer regulatory relationships, GrID-Net employs a modified GNN architecture with lagged message-passing, which enables it to capture the temporal dependencies between genomic loci and gene expression levels. By integrating temporal information with the graph-based model, GrID-Net offers a sophisticated approach to uncovering causative interactions in cellular regulatory networks, thereby contributing to a deeper understanding of gene regulation and its implications in complex biological processes.

**Biological network inference.** To infer disease-related biological associations, CIPHER-SC [80] leverages a variety of data sources including single-cell transcriptome data, protein-protein interaction (PPI), human phenotype ontology (HPO), and Online Mendelian Inheritance in Man (OMIM) to construct a heterogeneous graph, where nodes consist of diseases, genes, HPO terms and so on. CIPHER-SC applies GCN on the heterogeneous graph to infer disease-gene association.

**Cell type deconvolution.** SD^2^ [81] randomly combines cells from scRNA-seq data to generate pseudo-ST spots and constructs a spot-spot graph in a manner similar to DSTG, but it additionally incorporates the dropout information. Categorizing by cell type, SD^2^ employs a semi-supervised GCN to classify spots, and the output probabilities are regarded as the proportions of cell types within each spot.

**Multiple SRT slices integration.** Integrating multiple spatial transcriptomics (ST) slices is crucial for understanding complex tissue architecture across different layers and providing a more comprehensive view of the tissue microenvironment. Next, we introduce two GNN-based methods that address this task, each focusing on aligning and integrating spatial data from multiple slices to enable holistic analysis.

STitch3D [82] considers the 3D alignment between SRT slices. It takes spatial location information from multiple 2D SRT tissue slices and a matched well-labeled scRNA-seq dataset as inputs, and aligns 2D slices to form 3D spatial location. Treating each spot as a node, STitch3D constructs a global 3D neighborhood graph where nodes are connected if their 3D distance is less than 1.1 times the distance between the closest nodes within a single slice. It then employs a GTN-based model to learn integrated spot representations incorporating 3D global spatial information and cell type proportions based on these representations. The learned latent embeddings are also used for spatial domain identification across all slices via clustering algorithms and support various downstream analyses, including trajectory inference. According to the authors, Stitch3D demonstrated superior spatial clustering performance than SpaGCN and STAGATE.

SPACEL [83] also facilitates 3D alignment across multiple SRT slices. Initially, SPACEL utilizes annotated scRNA-seq data from the same tissue as SRT data to simulate SRT data, thereby estimating the cell type composition for spot-resolution SRT data. For single-cell level SRT data, SPACEL clusters cells and identifies cell types for each cluster through marker genes. Subsequently, SPACEL constructs a cell-cell or spot-spot graph based on their spatial location for each SRT slice and employs a GCN-based model incorporating an adversarial learning strategy to jointly learn representations of multiple slices, integrating the earlier derived cell type proportions. It then applies *k*-means clustering to identify spatial domains. Finally, SPACEL aligns the consecutive slices in a 3D manner based on previously obtained spatial domains.

**Protein abundance prediction.** Predicting protein abundance is a critical task in understanding cellular functions since proteins are the key executors of biological activities. Single-cell technologies like CITE-seq combine gene expression data with surface protein measurements, providing an opportunity to predict protein abundance based on gene expression and PPI networks.

For example, Dai *et al.* [84] introduced PIKE-R2P, a GNN-based approach that integrates prior biological knowledge from the PPI network to predict protein abundance from scRNA-seq data. The model constructs a graph with nodes representing genes and proteins, and edges capturing known interactions derived from the PPI network. A GNN is applied to this graph to predict protein levels, effectively incorporating prior knowledge about biological pathways and interactions. PIKE-R2P’s performance was validated on CITE-seq data, demonstrating its ability to predict surface protein abundance.

scMoFormer [85] takes a different approach by constructing a heterogeneous graph with nodes representing cells, genes, and proteins. It uses multimodal graph transformers to capture the relationships between these entities and predict the abundance of surface proteins at the single-cell level. By integrating multiple data types, scMoFormer effectively learns the complex interplay between gene expression and protein abundance.

**Cell-wise fluxome estimation.** Cell-wise fluxome estimation aims to quantify the flow of metabolites through metabolic pathways at the single-cell level. Fluxomics focuses on mapping the rate of biochemical reactions and how metabolites are produced and consumed within each cell. Estimating these fluxes offers valuable insights into cellular activities that are not fully captured by gene expression data alone, such as energy production efficiency and metabolic adaptation. Next, we introduce a GNN-based method developed to estimate cell-wise fluxomes by integrating transcriptomic and metabolic data.

scFEA [86] constructs a directed factor graph from the metabolic map, where nodes represent either variable nodes (reactions) or factor nodes (metabolites). Edges indicate the involvement of metabolites as substrates or products in specific reactions. This representation captures the complex relationships within the metabolic network. scFEA then applies a GNN on this factor graph, using scRNA-seq data to predict cell-wise metabolic fluxes at a single-cell level. By leveraging the structural representation of metabolism and integrating it with gene expression data, scFEA provides detailed insights into cell-wise metabolic flux at single-cell level.

**Text S14. The principle framework on how to apply GNNs to single-cell omics**

In this section, we have summarized and outlined the paradigms of applying GNNs to single-cell omics based on our review of 107 applications of GNNs across various single-cell omics. This includes problem definition, data collection and preprocessing, graph construction from single-cell data, GNN architecture selection, model training and optimization, and downstream task analysis. Below, we elaborate on each component.

First, it is essential to clearly define the problem at hand. This involves specifying which single-cell omics data will be used, the characteristics of the data (e.g., data from specific species, samples from individuals with particular diseases, or samples under specific perturbation conditions), and the target tasks to be solved with the data, such as cell clustering or classification, label transfer, data imputation, or gene regulatory network inference.

Second, data collection and preprocessing. According to our survey, the successful applications of GNNs in single-cell omics typically involve data from different species, technologies, and conditions (often including count matrices), as well as prior knowledge required for specific tasks, such as gene-gene interaction networks, protein-protein interaction (PPI) networks, and cell attributes (e.g., cell type). After data collection, preprocessing steps are commonly applied to count matrices, such as feature selection, normalization, and dimensionality reduction, to select highly variable features (e.g., genes) and reduce the dimensionality. This helps mitigate the challenges posed by high-dimensional and sparse characteristics of single-cell omics data.

Third, represent single-cell data as graph structures, where nodes can represent cells, genes, peaks, proteins, etc., and edges represent relationships between nodes. We have summarized the graph structures of each method in the 107 successful applications of GNNs in single-cell omics and presented them in the “Graph” column of Tables 2, 3, and 4 in the manuscript and Supplementary Table S1, and S2 of the supplementary file. There are many ways to construct graphs, such as building cell-cell graphs based on cell similarity (e.g., gene expression cosine similarity between cells), constructing cell-cell or spot-spot graphs based on spatial proximity, constructing gene-gene graphs based on gene association networks (e.g., gene regulatory network or gene co-expression network), constructing cell-gene graphs based on the gene expression matrix, and constructing cell-peak graphs based on cell-peak interactions. Additionally, appropriate feature engineering is required to encode node and edge attributes.

Fourth, selecting the appropriate GNN architecture based on the task requirements. Researchers can choose the suitable GNN architecture based on whether the task is transductive or inductive learning, the level of the task (graph-level tasks, such as graph classification; edge-level tasks, such as edge prediction or edge classification; or node-level tasks, such as node classification), and the scale of the graph constructed. This includes but is not limited to, the six GNN architectures primarily reviewed in our manuscript.

Fifth, model training and optimization employs standard machine learning workflows which generally include partitioning datasets into training, validation, and test sets, design of the task-specific loss function, performance evaluation, model architecture adjustment, and hyperparameter tuning.

Finally, downstream tasks are performed to assess whether the output of the model effectively addresses the target task and provides biological interpretability.

**Text S15. Explanation on the collection of commonly used single-cell datasets in GNN-based approaches**

In this section, we conducted a comprehensive summary of all 107 articles included in our survey to provide readers with practical resources for their own studies. Through systematic categorization by omics type, we collected and categorized 77 publicly available single-cell omics datasets which are commonly used in GNN-based single-cell studies. Below, we provide a detailed explanation of how we collected these widely used and publicly available single-cell omics datasets.

For single-cell epigenomics data, there are relatively few GNN-based methods specifically designed for this type of data. Therefore, we have compiled a list of nearly all datasets used by the reviewed methods in our survey that are focused on single-cell epigenomics. This compilation serves as a comprehensive resource for readers interested in conducting related research. Among these, the Cusanovich_2018 and Satpathy_2019 datasets were used twice. We have summarized the protocol information, species details, publication years, and sources for these 13 datasets in Supplementary Table S3.

In the case of single-cell transcriptomics data, due to the large number of methods available for this type of data, and the extensive datasets used by these methods, we presented 11 single-cell transcriptomics datasets that are widely used by GNN-based approaches for single-cell omics. Each of these datasets has been used in at least two of the articles reviewed in our paper. Among them, the Klein_2015 dataset has been used in at least seven articles, and the ZEISEL_2015 dataset has been used in at least five. We have summarized the species information, publication years, and sources for these 11 datasets in Supplementary Table S4.

Regarding spatially resolved transcriptomics data, there are also many GNN-based methods tailored for this type of data. Therefore, we presented 11 representative datasets that were widely used across multiple articles in the 107 studies reviewed in our paper. Among them, the Maynard_2021 dataset was used in at least nine studies, and the Stickels_2020 dataset was used in at least four studies. We have summarized the species information, publication years, and sources for these datasets in Supplementary Table S5.

We have compiled the 10 main single-cell proteomics datasets used in the articles we reviewed into Supplementary Table S6.

For single-cell multi-omics data, we have compiled 32 representative single-cell multi-omics datasets used in the 107 methods discussed in our paper. Given the diversity of technologies employed to obtain these datasets, we have also provided technology information for reference. The technology, species information, publication years, and sources for these datasets are summarized in Supplementary Table S7.

**Supplementary Tables**

**Table S1. Details of GNN-based methods for single-cell epigenomics.**

| **Algorithm name** | **Model** | **Graph** | **Year** | **Task type** | **Reference** |
| --- | --- | --- | --- | --- | --- |
| scGCN | GCNs | Cell-cell | 2021 | Label transfer | [14] |
| HyGAnno | VGAEs | Cell-cell | 2023 | Cell type identification | [87] |
| SANGO | GTNs | Cell-cell | 2024 | Cell type identification | [88] |
| DeepTFni | VGAEs | Gene-gene | 2022 | Gene regulatory network inference | [38] |
| GraphCpG | GCNs | Cell-locus | 2023 | Imputation | [89] |
| HiC-SGL | GNNs | Chromosomal segment-chromosomal segment | 2023 | Imputation | [90] |
| scHiMe | GTNs | Promoter-promoter | 2023 | Single-cell DNA methylation level prediction | [40] |

**Table S2. Details of GNN-based methods for single-cell proteomics.**

| **Algorithm name** | **Model** | **Graph** | **Year** | **Task type** | **Reference** |
| --- | --- | --- | --- | --- | --- |
| SNOWFLAKE | GNNs | Cell-cell | 2024 | Immune response prediction | [39] |
| scPROTEIN | GCNs | Cell-cell | 2024 | Dimensionality reduction | [91] |

**Table S3. Publicly available single-cell epigenomics datasets commonly used by 107 GNN-based methods in this review.**

| **Dataset** | **Technology** | **Species** | **Year** | **Reference** |
| --- | --- | --- | --- | --- |
| Cusanovich_2018 | sci-ATAC-seq | *Mus musculus* | 2018 | [92] |
| Fang_2021 | single-nucleus ATAC-seq | *Mus musculus* | 2021 | [93] |
| Preissl_2018 | single-nucleus ATAC-seq | *Homo sapiens; Mus musculus* | 2018 | [94] |
| Satpathy_2019 | scATAC-seq | *Homo sapiens; Mus musculus* | 2019 | [95] |
| Zhang_2021 | sci-ATAC-seq | *Homo sapiens* | 2021 | [96] |
| Hou_2016 | scRRBS-seq | *Homo sapiens; Mus musculus* | 2016 | [97] |
| Kretzmer_2021 | scRRBS-seq | *Homo sapiens* | 2021 | [98] |
| Farlik_2016 | scBS-seq | *Homo sapiens* | 2016 | [99] |
| Luo_2017 | snmC-seq | *Homo sapiens; Mus musculus* | 2017 | [100] |
| Ramani_2017 | scHi-C | *Homo sapiens; Mus musculus* | 2017 | [101] |
| Nagano_2017 | scHi-C | *Mus musculus* | 2017 | [102] |
| Kim_2020 | scHi-C | *Homo sapiens* | 2020 | [103] |
| Lee_2019 | sn-m3C-seq | *Homo sapiens; Mus musculus* | 2019 | [104] |

**Table S4. Publicly available single-cell transcriptomics datasets commonly used by 107 GNN-based methods in this review.**

| **Dataset** | **Species** | **Year** | **Reference** |
| --- | --- | --- | --- |
| Klein_2015 | *Homo sapiens; Mus musculus* | 2018 | [105] |
| ZEISEL_2015 | *Mus musculus* | 2015 | [106] |
| Chu_2016 | *Homo sapiens* | 2016 | [107] |
| Chung_2017 | *Homo sapiens* | 2017 | [108] |
| Baron_2016 | *Homo sapiens; Mus musculus* | 2016 | [109] |
| The Tabula Sapiens | *Homo sapiens* | 2022 | [110] |
| Frozen PBMCs (Donor A) | *Homo sapiens* | 2016 | [111] |
| Grubman_2019 | *Homo sapiens* | 2019 | [112] |
| Muraro_2016 | *Homo sapiens* | 2016 | [113] |
| Tabula Muris | *Mus musculus* | 2018 | [114] |
| Kim_2015 | *Mus musculus* | 2015 | [115] |

**Table S5. Publicly available single-cell spatially resolved transcriptomics datasets commonly used by 107 GNN-based methods in this review.**

| **Dataset** | **Species** | **Year** | **Reference** |
| --- | --- | --- | --- |
| He_2022 | *Homo sapiens; Mus musculus* | 2022 | [8] |
| Maynard_2021 | *Homo sapiens* | 2021 | [116] |
| Stickels_2020 | *Mus musculus* | 2020 | [117] |
| MOFFITT_2018 | *Mus musculus* | 2018 | [118] |
| Chen_2022 | *Mus musculus* | 2022 | [119] |
| WANG_2018 | *Mus musculus* | 2018 | [120] |
| Zhu_2018 | *Mus musculus* | 2018 | [121] |
| Xia_2019 | *Homo sapiens* | 2019 | [122] |
| Eng_2019 | *Mus musculus* | 2019 | [123] |
| Zhang_2021 | *Mus musculus* | 2021 | [124] |
| Wu_2021 | *Homo sapiens* | 2021 | [125] |

**Table S6. Publicly available single-cell proteomics datasets commonly used by 107 GNN-based methods in this review.**

| **Dataset** | **Species** | **Year** | **Reference** |
| --- | --- | --- | --- |
| SCoPE2_Specht dataset | *Homo sapiens* | 2021 | [126] |
| nanoPOTS dataset | *Homo sapiens* | 2019 | [127] |
| N2 dataset | *Mus musculus* | 2021 | [128] |
| SCoPE2_Leduc dataset | *Homo sapiens* | 2022 | [129] |
| pSCoPE_Leduc dataset | *Homo sapiens* | 2022 | [129] |
| plexDI dataset | *Homo sapiens* | 2022 | [130] |
| pSCoPE_Huffman dataset | *Homo sapiens* | 2023 | [131] |
| ECCITE-seq dataset | *Homo sapiens* | 2019 | [132] |
| BaselTMA dataset | *Homo sapiens* | 2020 | [133] |
| T-SCP dataset | *Homo sapiens* | 2022 | [134] |

**Table S7. Publicly available single-cell multi-omics datasets commonly used by 107 GNN-based methods in this review.**

| **Dataset** | **Technology** | **Species** | **Year** | **Reference** |
| --- | --- | --- | --- | --- |
| Chen_2019 | SNARE-seq | *Mus musculus* | 2019 | [135] |
| Ma_2020 | SHARE-seq | *Mus musculus* | 2020 | [136] |
| Saunders_2018 | Drop-seq | *Mus musculus* | 2018 | [137] |
| Luo_2017 | snmC-seq | *Mus musculus* | 2017 | [100] |
| CAO_2020 | sci-RNA-seq3 | *Homo sapiens* | 2020 | [138] |
| DOMCKE_2020 | sci-ATAC-seq3 | *Homo sapiens* | 2020 | [139] |
| Muto_2021 | snRNA-seq, snATAC- seq | *Homo sapiens* | 2021 | [140] |
| Yao_2021 | scRNA 10x v3, snATAC-seq | *Mus musculus* | 2021 | [141] |
| Muraro_2016 | Cel-seq2 | *Homo sapiens* | 2016 | [113] |
| Lawlor_2017 | Fluidigm C1 | *Homo sapiens* | 2017 | [142] |
| Baron_2016 | scRNA-seq | *Homo sapiens; Mus musculus* | 2016 | [109] |
| Hao_2021 | CITE-seq | *Homo sapiens; Mus musculus* | 2021 | [143] |
| Zheng_2017 | scRNA-seq | *Homo sapiens; Mus musculus* | 2017 | [111] |
| Maynard_2021 | Visium | *Homo sapiens* | 2021 | [116] |
| Nagy_2020 | 10x Genomics Chromium | *Homo sapiens* | 2020 | [144] |
| Eng_2019 | seqFISH+ | *Mus musculus* | 2019 | [123] |
| Tasic_2018 | SMART-seq | *Mus musculus* | 2018 | [145] |
| WANG_2018 | STARmap | *Mus musculus* | 2018 | [120] |
| MOFFITT_2018 | MERFISH | *Mus musculus* | 2018 | [118] |
| ORTIZ_2020 | ST | *Mus musculus* | 2020 | [146] |
| Kleshchevnikov_2022 | 10x Genomics Chromium | *Mus musculus* | 2022 | [147] |
| Asp_2019 | 10x Genomics Chromium | *Homo sapiens* | Asp | [148] |
| Lopez_2022 | 10x Genomics Chromium | *Mus musculus* | 2022 | [149] |
| Foster_2021 | Visium | *Mus musculus* | 2021 | [150] |
| Haensel_2020 | 10x Genomics Chromium | *Mus musculus* | 2020 | [151] |
| Andersson_2021 | ST | *Homo sapiens* | 2021 | [152] |
| Wu_2021 | 10x Genomics Chromium | *Homo sapiens* | 2021 | [125] |
| Wang_2022 | Stereo-seq | *Drosophila* | 2022 | [153] |
| CALDERON_2022 | sci-RNA-seq | *Drosophila* | 2022 | [154] |
| Tepe_2018 | scRNA-seq | *Mus musculus* | 2018 | [155] |
| Zeng_2023 | STARmap PLUS | *Mus musculus* | 2023 | [156] |
| CAO_2018 | sci-CAR | *Homo sapiens; Mus musculus* | 2018 | [157] |

**References**

1. Yuan Y, Bar-Joseph Z. GCNG: graph convolutional networks for inferring gene interaction from spatial transcriptomics data. Genome Biol 2020;21:1-16.

2. Dries R, Zhu Q, Dong R et al. Giotto: a toolbox for integrative analysis and visualization of spatial expression data. Genome Biol 2021;22:78.

3. Liu X, Shen Q, Zhang S. Cross-species cell-type assignment from single-cell RNA-seq data by a heterogeneous graph neural network. Genome Res 2023;33:96-111.

4. Wang Y, Wang R, Zhang S et al. iTALK: an R Package to Characterize and Illustrate Intercellular Communication. bioRxiv 2019:507871.

5. Raredon MSB, Yang J, Garritano J et al. Computation and visualization of cell–cell signaling topologies in single-cell systems data using Connectome. Sci Rep 2022;12:4187.

6. Jin S, Guerrero-Juarez CF, Zhang L et al. Inference and analysis of cell-cell communication using CellChat. Nat Commun 2021;12:1088.

7. Efremova M, Vento-Tormo M, Teichmann SA et al. CellPhoneDB: inferring cell–cell communication from combined expression of multi-subunit ligand–receptor complexes. Nat Protoc 2020;15:1484-1506.

8. He S, Bhatt R, Brown C et al. High-plex imaging of RNA and proteins at subcellular resolution in fixed tissue by spatial molecular imaging. Nat Biotechnol 2022;40:1794-1806.

9. Mohammadi S, Davila-Velderrain J, Kellis M. Reconstruction of Cell-type-Specific Interactomes at Single-Cell Resolution. Cell Systems 2019;9:559-568.e554.

10. Wang Y, Cho D-Y, Lee H et al. Reprogramming of regulatory network using expression uncovers sex-specific gene regulation in Drosophila. Nat Commun 2018;9:4061.

11. Kamimoto K, Stringa B, Hoffmann CM et al. Dissecting cell identity via network inference and in silico gene perturbation. Nature 2023;614:742-751.

12. Moerman T, Aibar Santos S, Bravo González-Blas C et al. GRNBoost2 and Arboreto: efficient and scalable inference of gene regulatory networks. Bioinformatics 2018;35:2159-2161.

13. Shu H, Zhou J, Lian Q et al. Modeling gene regulatory networks using neural network architectures. Nat Comput Sci 2021;1:491-501.

14. Song Q, Su J, Zhang W. scGCN is a graph convolutional networks algorithm for knowledge transfer in single cell omics. Nat Commun 2021;12:3826.

15. Stuart T, Butler A, Hoffman P et al. Comprehensive Integration of Single-Cell Data. Cell 2019;177:1888-1902.e1821.

16. Wang J, Ma A, Chang Y et al. scGNN is a novel graph neural network framework for single-cell RNA-Seq analyses. Nat Commun 2021;12:1882.

17. Li WV, Li JJ. An accurate and robust imputation method scImpute for single-cell RNA-seq data. Nat Commun 2018;9:997.

18. Lopez R, Regier J, Cole MB et al. Deep generative modeling for single-cell transcriptomics. Nat Methods 2018;15:1053-1058.

19. van der Maaten L, Hinton G. Visualizing Data using t-SNE. Journal of Machine Learning Research 2008;9:2579-2605.

20. Luo Z, Xu C, Zhang Z et al. A topology-preserving dimensionality reduction method for single-cell RNA-seq data using graph autoencoder. Sci Rep 2021;11:20028.

21. Amodio M, van Dijk D, Srinivasan K et al. Exploring single-cell data with deep multitasking neural networks. Nat Methods 2019;16:1139-1145.

22. Szubert B, Cole JE, Monaco C et al. Structure-preserving visualisation of high dimensional single-cell datasets. Sci Rep 2019;9:8914.

23. Moon KR, van Dijk D, Wang Z et al. Visualizing structure and transitions in high-dimensional biological data. Nat Biotechnol 2019;37:1482-1492.

24. Eraslan G, Simon LM, Mircea M et al. Single-cell RNA-seq denoising using a deep count autoencoder. Nat Commun 2019;10:390.

25. Wang T, Wu DJ, Coates A et al. End-to-end text recognition with convolutional neural networks. In: Proceedings of the 21st International Conference on Pattern Recognition (ICPR2012). 2012, p. 3304-3308. IEEE.

26. Krizhevsky A, Sutskever I, Hinton GE. Imagenet classification with deep convolutional neural networks. Advances in Neural Information Processing Systems 2012;25.

27. Ciresan DC, Meier U, Masci J et al. Flexible, high performance convolutional neural networks for image classification. In: Twenty-Second International Joint Conference on Artificial Intelligence. 2011. Citeseer.

28. Simonyan K, Zisserman A. Two-stream convolutional networks for action recognition in videos. Advances in Neural Information Processing Systems 2014;27.

29. Bruna J, Zaremba W, Szlam A et al. Spectral networks and deep locally connected networks on graphs. In: 2nd International Conference on Learning Representations, ICLR 2014. 2014.

30. Kipf TN, Welling M. Semi-Supervised Classification with Graph Convolutional Networks. International Conference on Learning Representations. 2017.

31. Hammond DK, Vandergheynst P, Gribonval R. Wavelets on graphs via spectral graph theory. Appl Comput Harmon Anal 2011;30:129-150.

32. Hamilton W, Ying Z, Leskovec J. Inductive representation learning on large graphs. Advances in Neural Information Processing Systems. 2017.

33. Veličković P, Cucurull G, Casanova A et al. Graph Attention Networks. International Conference on Learning Representations. 2018.

34. Yun S, Jeong M, Kim R et al. Graph transformer networks. Advances in neural information processing systems 2019;32.

35. Kipf TN, Welling M. Variational graph auto-encoders. arXiv preprint arXiv:1611.07308 2016.

36. Gilmer J, Schoenholz SS, Riley PF et al. Neural message passing for quantum chemistry. In: International conference on machine learning. 2017, p. 1263-1272. PMLR.

37. Gilmer J, Schoenholz SS, Riley PF et al. Message passing neural networks. Machine learning meets quantum physics 2020:199-214.

38. Li H, Sun Y, Hong H et al. Inferring transcription factor regulatory networks from single-cell ATAC-seq data based on graph neural networks. Nat Mach Intell 2022;4:389-400.

39. Hu T, Allam M, Kaushik V et al. Spatial Morphoproteomic Features Predict Uniqueness of Immune Microarchitectures and Responses in Lymphoid Follicles. bioRxiv 2024.

40. Zhu H, Liu T, Wang Z. scHiMe: predicting single-cell DNA methylation levels based on single-cell Hi-C data. Brief Bioinform 2023;24:bbad223.

41. Xiong Z, Luo J, Shi W et al. scGCL: an imputation method for scRNA-seq data based on graph contrastive learning. Bioinformatics 2023;39.

42. Lee N, Lee J, Park C. Augmentation-free self-supervised learning on graphs. In: Proceedings of the AAAI Conference on Artificial Intelligence. 2022, p. 7372-7380.

43. Buterez D, Bica I, Tariq I et al. CellVGAE: an unsupervised scRNA-seq analysis workflow with graph attention networks. Bioinformatics 2022;38:1277-1286.

44. McInnes L, Healy J, Astels S. hdbscan: Hierarchical density based clustering. J. Open Source Softw. 2017;2:205.

45. Gu H, Cheng H, Ma A et al. scGNN 2.0: a graph neural network tool for imputation and clustering of single-cell RNA-Seq data. Bioinformatics 2022;38:5322-5325.

46. Feng X, Xiu Y-H, Long H-X et al. Advancing single-cell RNA-seq data analysis through the fusion of multi-layer perceptron and graph neural network. Brief Bioinform 2024;25:bbad481.

47. Ji C, Yu N, Wang Y et al. An end-to-end Deep Hybrid Autoencoder based method for single-cell RNA-Seq data analysis. IEEE/ACM Trans Comput Biol Bioinform 2023.

48. Tian S-W, Ni J-C, Wang Y-T et al. scgcc: Graph contrastive clustering with neighborhood augmentations for scrna-seq data analysis. IEEE J Biomed Health Inf 2023.

49. Wang T, Bai J, Nabavi S. Single-cell classification using graph convolutional networks. BMC Bioinformatics 2021;22:1-23.

50. Yang R, Dai W, Li C et al. scBiGNN: Bilevel Graph Representation Learning for Cell Type Classification from Single-cell RNA Sequencing Data. NeurIPS 2023 AI for Science Workshop. 2023.

51. Hu D, Guan R, Liang K et al. scEGG: an exogenous gene-guided clustering method for single-cell transcriptomic data. Brief Bioinform 2024;25.

52. Shao X, Yang H, Zhuang X et al. scDeepSort: a pre-trained cell-type annotation method for single-cell transcriptomics using deep learning with a weighted graph neural network. Nucleic Acids Res 2021;49:e122-e122.

53. Yuan M, Chen L, Deng M. scMRA: a robust deep learning method to annotate scRNA-seq data with multiple reference datasets. Bioinformatics 2022;38:738-745.

54. Zeng Y, Wei Z, Pan Z et al. A robust and scalable graph neural network for accurate single-cell classification. Brief Bioinform 2022;23:bbab570.

55. Wang YM, Sun Y, Wang B et al. Transfer learning for clustering single-cell RNA-seq data crossing-species and batch, case on uterine fibroids. Brief Bioinform 2024;25:bbad426.

56. Cheng Y, Ma X. scGAC: a graph attentional architecture for clustering single-cell RNA-seq data. Bioinformatics 2022;38:2187-2193.

57. Li S, Guo H, Zhang S et al. Attention-based deep clustering method for scRNA-seq cell type identification. PLoS Comput Biol 2023;19:e1011641.

58. Wang L, Li W, Xie W et al. Dual-GCN-based deep clustering with triplet contrast for ScRNA-seq data analysis. Comput Biol Chem 2023;106:107924.

59. Mao G, Pang Z, Zuo K et al. Predicting gene regulatory links from single-cell RNA-seq data using graph neural networks. Brief Bioinform 2023;24:bbad414.

60. Yang Y, Li G, Zhong Y et al. Gene knockout inference with variational graph autoencoder learning single-cell gene regulatory networks. Nucleic Acids Res 2023;51:6578-6592.

61. Osorio D, Zhong Y, Li G et al. scTenifoldNet: a machine learning workflow for constructing and comparing transcriptome-wide gene regulatory networks from single-cell data. Patterns 2020;1.

62. Ravindra N, Sehanobish A, Pappalardo JL et al. Disease state prediction from single-cell data using graph attention networks. In: ACM Conference on Health, Inference, and Learning. 2020, p. 121-130.

63. Sehanobish A, Ravindra N, van Dijk D. Gaining insight into sars-cov-2 infection and COVID-19 severity using self-supervised edge features and graph neural networks. In: Proceedings of the AAAI Conference on Artificial Intelligence. 2021, p. 4864-4873.

64. Ying Z, Bourgeois D, You J et al. Gnnexplainer: Generating explanations for graph neural networks. Advances in Neural Information Processing Systems 2019;32.

65. Lee J, Lee Y, Kim J et al. Set transformer: A framework for attention-based permutation-invariant neural networks. In: International Conference on Machine Learning. 2019, p. 3744-3753. PMLR.

66. Roohani Y, Huang K, Leskovec J. Predicting transcriptional outcomes of novel multigene perturbations with GEARS. Nat Biotechnol 2024;42:927-935.

67. Bunne C, Stark SG, Gut G et al. Learning single-cell perturbation responses using neural optimal transport. Nat Methods 2023;20:1759-1768.

68. Shahir JA, Stanley N, Purvis JE. Cellograph: a semi-supervised approach to analyzing multi-condition single-cell RNA-sequencing data using graph neural networks. BMC Bioinformatics 2024;25:25.

69. Li Z, Chen X, Zhang X et al. Latent feature extraction with a prior-based self-attention framework for spatial transcriptomics. Genome Res 2023;33:1757-1773.

70. Ren H, Walker BL, Cang Z et al. Identifying multicellular spatiotemporal organization of cells with SpaceFlow. Nat Commun 2022;13:4076.

71. Velickovic P, Fedus W, Hamilton WL et al. Deep graph infomax. ICLR (Poster) 2019;2:4.

72. Haghverdi L, Büttner M, Wolf FA et al. Diffusion pseudotime robustly reconstructs lineage branching. Nat Methods 2016;13:845-848.

73. Li J, Chen S, Pan X et al. Cell clustering for spatial transcriptomics data with graph neural networks. Nat Comput Sci 2022;2:399-408.

74. Long Y, Ang KS, Li M et al. Spatially informed clustering, integration, and deconvolution of spatial transcriptomics with GraphST. Nat Commun 2023;14:1155.

75. Fischer DS, Schaar AC, Theis FJ. Modeling intercellular communication in tissues using spatial graphs of cells. Nat Biotechnol 2023;41:332-336.

76. Tang Z, Zhang T, Yang B et al. spaCI: deciphering spatial cellular communications through adaptive graph model. Brief Bioinform 2023;24:bbac563.

77. Zhang X, Wang X, Shivashankar G et al. Graph-based autoencoder integrates spatial transcriptomics with chromatin images and identifies joint biomarkers for Alzheimer’s disease. Nat Commun 2022;13:7480.

78. Liu Y, Zhang J, Wang S et al. A heterogeneous graph cross-omics attention model for single-cell representation learning. In: IEEE International Conference on Bioinformatics and Biomedicine. 2022, p. 270-275. IEEE.

79. Gao H, Zhang B, Liu L et al. A universal framework for single-cell multi-omics data integration with graph convolutional networks. Brief Bioinform 2023;24:bbad081.

80. Zhang Y, Chen L, Li S. CIPHER-SC: disease-gene association inference using graph convolution on a context-aware network with single-cell data. IEEE/ACM Trans Comput Biol Bioinform 2020;19:819-829.

81. Li H, Li H, Zhou J et al. SD2: spatially resolved transcriptomics deconvolution through integration of dropout and spatial information. Bioinformatics 2022;38:4878-4884.

82. Wang G, Zhao J, Yan Y et al. Construction of a 3D whole organism spatial atlas by joint modelling of multiple slices with deep neural networks. Nat Mach Intell 2023;5:1200-1213.

83. Xu H, Wang S, Fang M et al. SPACEL: deep learning-based characterization of spatial transcriptome architectures. Nat Commun 2023;14:7603.

84. Dai X, Xu F, Wang S et al. PIKE-R2P: Protein–protein interaction network-based knowledge embedding with graph neural network for single-cell RNA to protein prediction. BMC Bioinformatics 2021;22:139.

85. Tang W, Wen H, Liu R et al. Single-cell multimodal prediction via transformers. In: Proceedings of the 32nd ACM International Conference on Information and Knowledge Management. 2023, p. 2422-2431.

86. Alghamdi N, Chang W, Dang P et al. A graph neural network model to estimate cell-wise metabolic flux using single-cell RNA-seq data. Genome Res 2021;31:1867-1884.

87. Zhang W, Cui Y, Liu B et al. HyGAnno: hybrid graph neural network–based cell type annotation for single-cell ATAC sequencing data. Brief Bioinform 2024;25:bbae152.

88. Zeng Y, Luo M, Shangguan N et al. Deciphering cell types by integrating scATAC-seq data with genome sequences. Nat Comput Sci 2024:1-14.

89. Deng Y, Tang J, Zhang J et al. GraphCpG: imputation of single-cell methylomes based on locus-aware neighboring subgraphs. Bioinformatics 2023;39:btad533.

90. Zheng J, Yang Y, Dai Z. Subgraph extraction and graph representation learning for single cell Hi-C imputation and clustering. Brief Bioinform 2024;25:bbad379.

91. Li W, Yang F, Wang F et al. scPROTEIN: a versatile deep graph contrastive learning framework for single-cell proteomics embedding. Nat Methods 2024;21:623-634.

92. Cusanovich DA, Hill AJ, Aghamirzaie D et al. A Single-Cell Atlas of In Vivo Mammalian Chromatin Accessibility. Cell 2018;174:1309-1324.e1318.

93. Fang R, Preissl S, Li Y et al. Comprehensive analysis of single cell ATAC-seq data with SnapATAC. Nat Commun 2021;12:1337.

94. Preissl S, Fang R, Huang H et al. Single-nucleus analysis of accessible chromatin in developing mouse forebrain reveals cell-type-specific transcriptional regulation. Nat Neurosci 2018;21:432-439.

95. Satpathy AT, Granja JM, Yost KE et al. Massively parallel single-cell chromatin landscapes of human immune cell development and intratumoral T cell exhaustion. Nat Biotechnol 2019;37:925-936.

96. Zhang K, Hocker JD, Miller M et al. A single-cell atlas of chromatin accessibility in the human genome. Cell 2021;184:5985-6001. e5919.

97. Hou Y, Guo H, Cao C et al. Single-cell triple omics sequencing reveals genetic, epigenetic, and transcriptomic heterogeneity in hepatocellular carcinomas. Cell Res 2016;26:304-319.

98. Kretzmer H, Biran A, Purroy N et al. Preneoplastic Alterations Define CLL DNA Methylome and Persist through Disease Progression and Therapy. Blood Cancer Discovery 2021;2:54-69.

99. Farlik M, Halbritter F, Müller F et al. DNA methylation dynamics of human hematopoietic stem cell differentiation. Cell Stem Cell 2016;19:808-822.

100. Luo C, Keown CL, Kurihara L et al. Single-cell methylomes identify neuronal subtypes and regulatory elements in mammalian cortex. Science 2017;357:600-604.

101. Ramani V, Deng X, Qiu R et al. Massively multiplex single-cell Hi-C. Nat Methods 2017;14:263-266.

102. Nagano T, Lubling Y, Várnai C et al. Cell-cycle dynamics of chromosomal organization at single-cell resolution. Nature 2017;547:61-67.

103. Kim H-J, Yardımcı GG, Bonora G et al. Capturing cell type-specific chromatin compartment patterns by applying topic modeling to single-cell Hi-C data. PLoS Comput Biol 2020;16:e1008173.

104. Lee D-S, Luo C, Zhou J et al. Simultaneous profiling of 3D genome structure and DNA methylation in single human cells. Nat Methods 2019;16:999-1006.

105. Klein Allon M, Mazutis L, Akartuna I et al. Droplet Barcoding for Single-Cell Transcriptomics Applied to Embryonic Stem Cells. Cell 2015;161:1187-1201.

106. Zeisel A, Muñoz-Manchado AB, Codeluppi S et al. Cell types in the mouse cortex and hippocampus revealed by single-cell RNA-seq. Science 2015;347:1138-1142.

107. Chu L-F, Leng N, Zhang J et al. Single-cell RNA-seq reveals novel regulators of human embryonic stem cell differentiation to definitive endoderm. Genome Biol 2016;17:173.

108. Chung W, Eum HH, Lee H-O et al. Single-cell RNA-seq enables comprehensive tumour and immune cell profiling in primary breast cancer. Nat Commun 2017;8:15081.

109. Baron M, Veres A, Wolock Samuel L et al. A Single-Cell Transcriptomic Map of the Human and Mouse Pancreas Reveals Inter- and Intra-cell Population Structure. Cell Systems 2016;3:346-360.e344.

110. Jones RC, Karkanias J, Krasnow MA et al. The Tabula Sapiens: A multiple-organ, single-cell transcriptomic atlas of humans. Science 2022;376:eabl4896.

111. Zheng GXY, Terry JM, Belgrader P et al. Massively parallel digital transcriptional profiling of single cells. Nat Commun 2017;8:14049.

112. Grubman A, Chew G, Ouyang JF et al. A single-cell atlas of entorhinal cortex from individuals with Alzheimer’s disease reveals cell-type-specific gene expression regulation. Nat Neurosci 2019;22:2087-2097.

113. Muraro Mauro J, Dharmadhikari G, Grün D et al. A Single-Cell Transcriptome Atlas of the Human Pancreas. Cell Systems 2016;3:385-394.e383.

114. Schaum N, Karkanias J, Neff NF et al. Single-cell transcriptomics of 20 mouse organs creates a Tabula Muris. Nature 2018;562:367-372.

115. Kim JK, Kolodziejczyk AA, Ilicic T et al. Characterizing noise structure in single-cell RNA-seq distinguishes genuine from technical stochastic allelic expression. Nat Commun 2015;6:8687.

116. Maynard KR, Collado-Torres L, Weber LM et al. Transcriptome-scale spatial gene expression in the human dorsolateral prefrontal cortex. Nat Neurosci 2021;24:425-436.

117. Stickels RR, Murray E, Kumar P et al. Highly sensitive spatial transcriptomics at near-cellular resolution with Slide-seqV2. Nat Biotechnol 2021;39:313-319.

118. Moffitt JR, Bambah-Mukku D, Eichhorn SW et al. Molecular, spatial, and functional single-cell profiling of the hypothalamic preoptic region. Science 2018;362:eaau5324.

119. Chen A, Liao S, Cheng M et al. Spatiotemporal transcriptomic atlas of mouse organogenesis using DNA nanoball-patterned arrays. Cell 2022;185:1777-1792.e1721.

120. Wang X, Allen WE, Wright MA et al. Three-dimensional intact-tissue sequencing of single-cell transcriptional states. Science 2018;361:eaat5691.

121. Zhu Q, Shah S, Dries R et al. Identification of spatially associated subpopulations by combining scRNAseq and sequential fluorescence in situ hybridization data. Nat Biotechnol 2018;36:1183-1190.

122. Xia C, Fan J, Emanuel G et al. Spatial transcriptome profiling by MERFISH reveals subcellular RNA compartmentalization and cell cycle-dependent gene expression. Proceedings of the National Academy of Sciences 2019;116:19490-19499.

123. Eng C-HL, Lawson M, Zhu Q et al. Transcriptome-scale super-resolved imaging in tissues by RNA seqFISH+. Nature 2019;568:235-239.

124. Zhang M, Eichhorn SW, Zingg B et al. Spatially resolved cell atlas of the mouse primary motor cortex by MERFISH. Nature 2021;598:137-143.

125. Wu SZ, Al-Eryani G, Roden DL et al. A single-cell and spatially resolved atlas of human breast cancers. Nat Genet 2021;53:1334-1347.

126. Specht H, Emmott E, Petelski AA et al. Single-cell proteomic and transcriptomic analysis of macrophage heterogeneity using SCoPE2. Genome Biol 2021;22:50.

127. Dou M, Clair G, Tsai C-F et al. High-Throughput Single Cell Proteomics Enabled by Multiplex Isobaric Labeling in a Nanodroplet Sample Preparation Platform. Anal Chem 2019;91:13119-13127.

128. Woo J, Williams SM, Markillie LM et al. High-throughput and high-efficiency sample preparation for single-cell proteomics using a nested nanowell chip. Nat Commun 2021;12:6246.

129. Leduc A, Huffman RG, Cantlon J et al. Exploring functional protein covariation across single cells using nPOP. Genome Biol 2022;23:261.

130. Derks J, Leduc A, Wallmann G et al. Increasing the throughput of sensitive proteomics by plexDIA. Nat Biotechnol 2023;41:50-59.

131. Huffman RG, Leduc A, Wichmann C et al. Prioritized mass spectrometry increases the depth, sensitivity and data completeness of single-cell proteomics. Nat Methods 2023;20:714-722.

132. Mimitou EP, Cheng A, Montalbano A et al. Multiplexed detection of proteins, transcriptomes, clonotypes and CRISPR perturbations in single cells. Nat Methods 2019;16:409-412.

133. Jackson HW, Fischer JR, Zanotelli VRT et al. The single-cell pathology landscape of breast cancer. Nature 2020;578:615-620.

134. Brunner AD, Thielert M, Vasilopoulou C et al. Ultra‐high sensitivity mass spectrometry quantifies single‐cell proteome changes upon perturbation. Mol Syst Biol 2022;18:e10798.

135. Chen S, Lake BB, Zhang K. High-throughput sequencing of the transcriptome and chromatin accessibility in the same cell. Nat Biotechnol 2019;37:1452-1457.

136. Ma S, Zhang B, LaFave LM et al. Chromatin Potential Identified by Shared Single-Cell Profiling of RNA and Chromatin. Cell 2020;183:1103-1116.e1120.

137. Saunders A, Macosko EZ, Wysoker A et al. Molecular Diversity and Specializations among the Cells of the Adult Mouse Brain. Cell 2018;174:1015-1030.e1016.

138. Cao J, O’Day DR, Pliner HA et al. A human cell atlas of fetal gene expression. Science 2020;370:eaba7721.

139. Domcke S, Hill AJ, Daza RM et al. A human cell atlas of fetal chromatin accessibility. Science 2020;370:eaba7612.

140. Muto Y, Wilson PC, Ledru N et al. Single cell transcriptional and chromatin accessibility profiling redefine cellular heterogeneity in the adult human kidney. Nat Commun 2021;12:2190.

141. Yao Z, Liu H, Xie F et al. A transcriptomic and epigenomic cell atlas of the mouse primary motor cortex. Nature 2021;598:103-110.

142. Lawlor N, George J, Bolisetty M et al. Single-cell transcriptomes identify human islet cell signatures and reveal cell-type-specific expression changes in type 2 diabetes. Genome Res 2017;27:208-222.

143. Hao Y, Hao S, Andersen-Nissen E et al. Integrated analysis of multimodal single-cell data. Cell 2021;184:3573-3587.e3529.

144. Nagy C, Maitra M, Tanti A et al. Single-nucleus transcriptomics of the prefrontal cortex in major depressive disorder implicates oligodendrocyte precursor cells and excitatory neurons. Nat Neurosci 2020;23:771-781.

145. Tasic B, Yao Z, Graybuck LT et al. Shared and distinct transcriptomic cell types across neocortical areas. Nature 2018;563:72-78.

146. Ortiz C, Navarro JF, Jurek A et al. Molecular atlas of the adult mouse brain. Science Advances 2020;6:eabb3446.

147. Kleshchevnikov V, Shmatko A, Dann E et al. Cell2location maps fine-grained cell types in spatial transcriptomics. Nat Biotechnol 2022;40:661-671.

148. Asp M, Giacomello S, Larsson L et al. A Spatiotemporal Organ-Wide Gene Expression and Cell Atlas of the Developing Human Heart. Cell 2019;179:1647-1660.e1619.

149. Lopez R, Li B, Keren-Shaul H et al. DestVI identifies continuums of cell types in spatial transcriptomics data. Nat Biotechnol 2022;40:1360-1369.

150. Foster DS, Januszyk M, Yost KE et al. Integrated spatial multiomics reveals fibroblast fate during tissue repair. Proceedings of the National Academy of Sciences 2021;118:e2110025118.

151. Haensel D, Jin S, Sun P et al. Defining Epidermal Basal Cell States during Skin Homeostasis and Wound Healing Using Single-Cell Transcriptomics. Cell Reports 2020;30:3932-3947.e3936.

152. Andersson A, Larsson L, Stenbeck L et al. Spatial deconvolution of HER2-positive breast cancer delineates tumor-associated cell type interactions. Nat Commun 2021;12:6012.

153. Wang M, Hu Q, Lv T et al. High-resolution 3D spatiotemporal transcriptomic maps of developing Drosophila embryos and larvae. Dev Cell 2022;57:1271-1283.e1274.

154. Calderon D, Blecher-Gonen R, Huang X et al. The continuum of <i>Drosophila</i> embryonic development at single-cell resolution. Science 2022;377:eabn5800.

155. Tepe B, Hill MC, Pekarek BT et al. Single-Cell RNA-Seq of Mouse Olfactory Bulb Reveals Cellular Heterogeneity and Activity-Dependent Molecular Census of Adult-Born Neurons. Cell Reports 2018;25:2689-2703.e2683.

156. Zeng H, Huang J, Zhou H et al. Integrative in situ mapping of single-cell transcriptional states and tissue histopathology in a mouse model of Alzheimer’s disease. Nat Neurosci 2023;26:430-446.

157. Cao J, Cusanovich DA, Ramani V et al. Joint profiling of chromatin accessibility and gene expression in thousands of single cells. Science 2018;361:1380-1385.
